# Synthesis, Structure, and Electronic Properties of [Cr_6_@Sn_8_Sb_8_(en)_2_]^3–^: A Cr_6_ Octahedron Encapsulated in a Zintl-Ion Ligand

**DOI:** 10.1021/jacs.5c00515

**Published:** 2025-07-08

**Authors:** Wei-Xing Chen, Aidan Manley, Zi-Sheng Li, John E. McGrady, Zhong-Ming Sun

**Affiliations:** † State Key Laboratory of Elemento-Organic Chemistry, Tianjin Key Lab for Rare Earth Materials and Applications, School of Materials Science and Engineering, 12538 Nankai University, Tianjin 300350, China; ‡ Department of Chemistry, 6396 University of Oxford, Oxford OX1 3QZ, U.K.

## Abstract

The reaction of CrCp_2_ with K_2_SnSb yields a Zintl-ion cluster containing an octahedral Cr_6_ core, surrounded by an Sn_8_Sb_8_ ring and two further ethylenediamine ligands that cap two mutually *trans* Cr centers. The Cr–Cr bond lengths of 2.453(2) to 2.482(2) Å are remarkably short, much shorter than those in any of the 20-electron chalcogenide-capped analogues, Cr_6_E_8_(PR_3_)_6_, indicating unusually strong Cr–Cr bonding. An isotropic signal in the electron paramagnetic resonance (EPR) spectrum with *g* = 2.21 is consistent with a 23-electron Cr_6_^13+^ core, three electrons more reduced than the Cr_6_^16+^ units present in Cr_6_E_8_(PR_3_)_6_. An analysis of the electronic structure using density functional theory (DFT) indicates that the additional three electrons enter Cr–Cr bonding orbitals, which are stabilized as a result of the weak π-donor properties of the Sn_8_Sb_8_^16–^ Zintl ligand.

## Graphical abstract

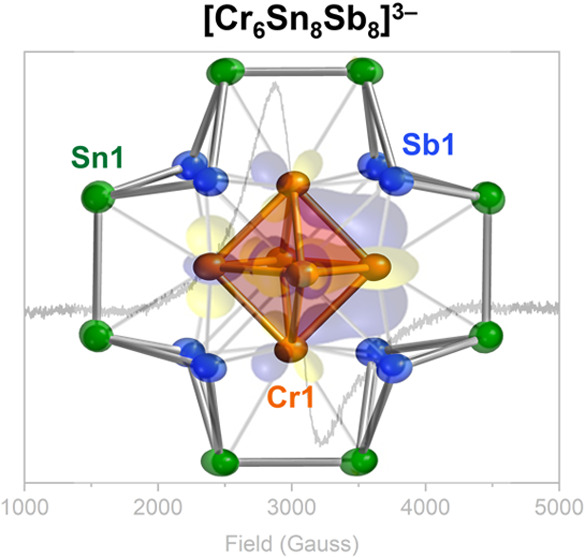

## Introduction

The elements of group 6, chromium molybdenum and tungsten, have played a central part in the history of the metal–metal bond,^1,2^ beginning with the first report of Cr_2_(CH_3_COO)_4_(H_2_O)_2_ by Peligot,^3^ a dimer that was shown, over a century later, to contain a Cr–Cr quadruple bond.^4,5^ More recently, the use of alkali metal reducing agents has given access to a range of Cr^I^ species linked by quintuple bonds, exemplified by Power’s 2005 discovery of *trans*-bent Cr_2_Ar'_2_, Ar' = C_6_H_3_-2,6-(C_6_H_3_-2,6-(CHMe_2_)_2_)_2_ (Figure 1a).^6,7^ Larger clusters containing three or more Cr atoms are also well established, notably in Betley’s trichromium complex (^tbs^L)­Cr_3_(thf), which shows promise in small molecule activation (Figure 1b).^8^ The coordination spheres of the clusters described above are completed by traditional inorganic and organometallic ligands such as amides, sulfides, phosphines, and alkyl groups, but alternative ligand architectures made up of circular or spherical arrays of *p*-block (semi)metal atoms such as Ge, Sn, As, Sb (“Zintl ions”) are emerging as alternative scaffolds for stabilizing metal ions and clusters.^9–11^ Notable recent examples include [Fe@Ge_10_]^3–^,^12^ [Ru@Ge_12_]^3–^,^13^ [Ln@Sb_12_]^3–^ (Ln = La, Y, Ho, Er, Lu),^14^ and [An@Bi_12_]^n–^ (An/n = U/3; Th/4)^15,16^ (Figure 1d), where we use @ to indicate endohedral encapsulation. The structures of the last two molecules in this list highlight the important principle that cyclic arrays made up of three butterfly Pn_4_ units can span the circumference of relatively large metal cations. There are rather fewer examples where a cluster of three or more transition metals, as distinct from an isolated atom, is encapsulated within a Zintl core, but the [Pd_3_Sn_8_Bi_6_]^4–^ cluster (Figure 1e) was reported by Dehnen and co-workers.^17^ In a very recent publication, we have shown that a low-valent Cr precursor, CrCp_2_, reacts with the Sb-rich Zintl phase K_8_SnSb_4_ to form a pentagonal Cr_5_ cluster, [Cr_5_Sb_20_Sn_2_]^4–^, with very short Cr–Cr bonds (2.539– 2.625 Å) encapsulated by an Sb_20_ ring made up of five fused Sb_4_ units.^18^ Extrapolating from [Ln@Sb_12_]^3–^, it seems that cyclic arrays based on Pn_4_ units (or their isoelectronic analogues) may therefore provide a versatile platform for encapsulating clusters of different sizes. Inspired by this hypothesis, we now report our attempts to use a more Sn-rich Zintl phase, K_2_SnSb, as a Pn_4_ surrogate to construct chromium cluster compounds. The pseudoelement concept establishes an equivalence between Sb and Sn^–^, so increasing Sn content should increase the net negative charge on the main group component, which may, in turn, stabilize a more highly oxidized Cr core than the one present in [Cr_5_Sb_20_Sn_2_]^4–^. We show here that the reaction of K_2_SnSb with CrCp_2_ leads to a new Zintl cluster, [Cr_6_@Sn_8_Sb_8_(en)_2_]^3–^ (Figure 1f), containing an octahedral Cr_6_ core encapsulated in a Zintl-ion ring made up of four fused Sn_2_Sb_2_ units. The Cr–Cr bond lengths are substantially shorter than those in the Cr_6_E_8_(PR_3_)_6_ family (Figure 1c), a well-established structural motif across the majority of the transition block.^19–26^ In addition to characterization through X-ray crystallography and electron paramagnetic resonance (EPR) spectroscopy, we use density functionals to explore the electronic structure of this new cluster and establish its place in the context of the extended octahedral M_6_ family.

**Figure 1:**
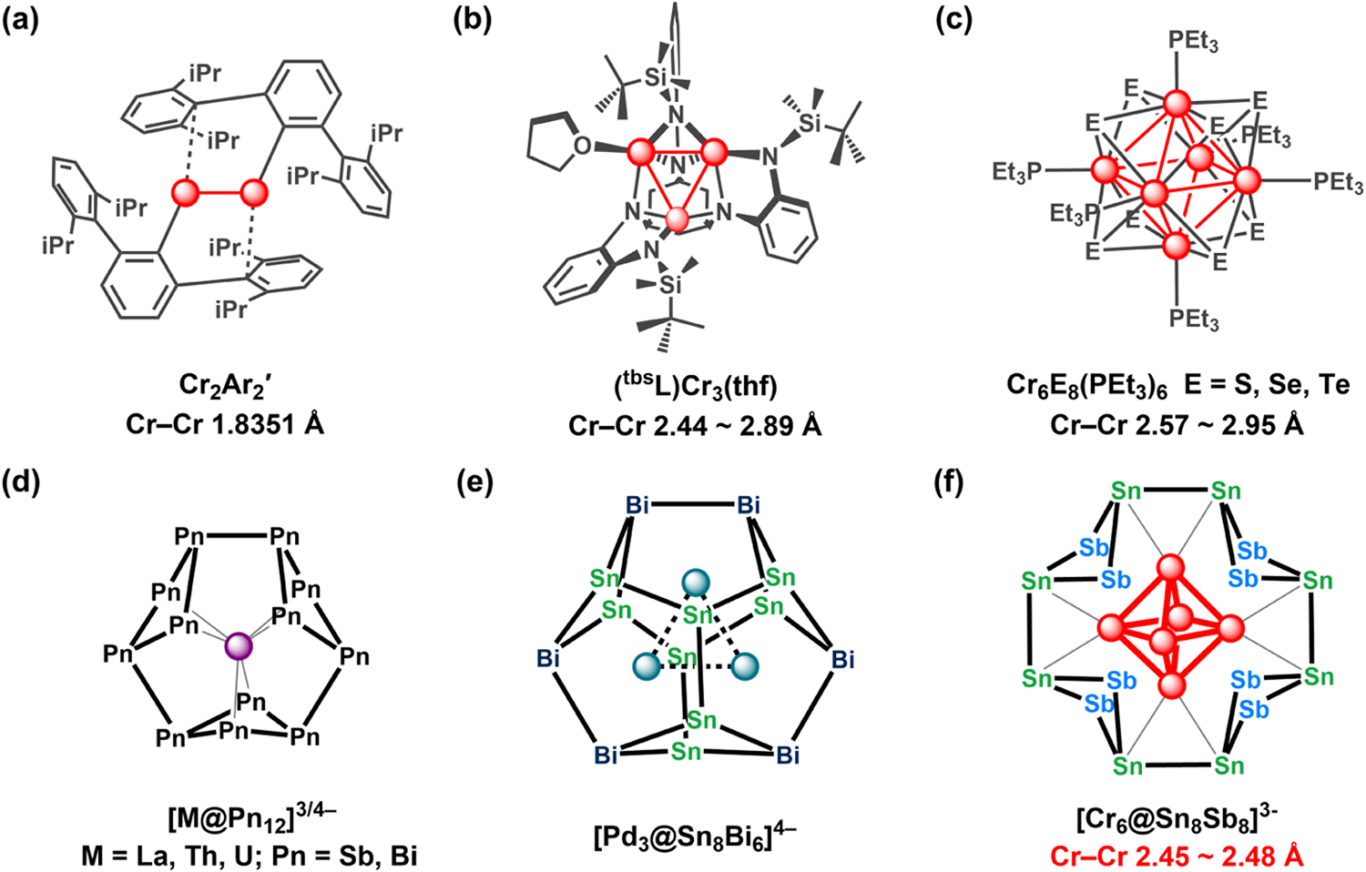
Examples of clusters stabilized by classic ligand sets (a–c) and by cyclic “Zintl” arrays of *p*-block elements (d–f).

## Results and Discussion

### Synthesis and Structure

{[K­(18-C-6)]_2_Cp}_3_{[Cr_6_@Sn_8_Sb_8_(en)_2_]} (**1**) was prepared by reacting an ethylenediamine (en) solution of the ternary Zintl precursor K_2_SnSb with CrCp_2_ in the presence of 18-crown-6 at room temperature. Full details of the synthesis are given in the Experimental Section. **1** crystallizes in the triclinic space group *P*1̅, and the unit cell contains a single [Cr_6_@Sn_8_Sb_8_(en)_2_]^3–^ anion as well as six K^+^ cations, three of which are bound to Cp^–^ ligands derived from the CrCp_2_ starting material (Figure 2a and Supporting Information, Figures S1–S3). Important bond lengths are collected in Table 1. The formulation of the cluster as a trianion, [Cr_6_@Sn_8_Sb_8_(en)_2_]^3–^, requires that it is paramagnetic, and indeed, the electron paramagnetic resonance (EPR) spectrum measured at 95 K, shown in Figure 3, shows a broad anisotropic signal centered at *g* = 2.21. The Cr_6_@Sn_8_Sb_8_ core of the cluster has approximate *D*_4*h*_ symmetry, with four Cr atoms in the equatorial plane surrounded by a Sn_8_Sb_8_ ring. The remaining two Cr atoms bind in the axial positions and are terminated by en ligands, which are bound via a single nitrogen atom. All 12 Cr–Cr bond lengths are below 2.5 Å, with those in the equatorial plane marginally shorter, at ∼2.46 Å, than those between equatorial and axial Cr atoms (∼2.48 Å). These values are intermediate between those in the recently characterized [Cr_5_Sn_2_Sb_20_]^4–^ cluster (2.59–2.62 Å)^18^ and the [Cr_2_Sb_12_]^3–^ dimer (2.319 Å), where significant Cr–Cr multiple bonding is present.^27^ The Cr–Cr bonds in **1** are also strikingly shorter than those in any of the Cr_6_ chalcogenides, Cr_6_E_8_(PR_3_)_6_ (also collected in Table 1).^19–26^ The most direct comparison is to the telluride, Cr_6_Te_8_(PR_3_)_6_ (because Te is in the same period as Sn and Sb), and there the average Cr–Cr bond length is in excess of 2.80 Å. The Cr–Cr bonding in **1** appears, therefore, to stand out as being much stronger than in comparable Cr_6_ octahedra.

**Figure 2:**
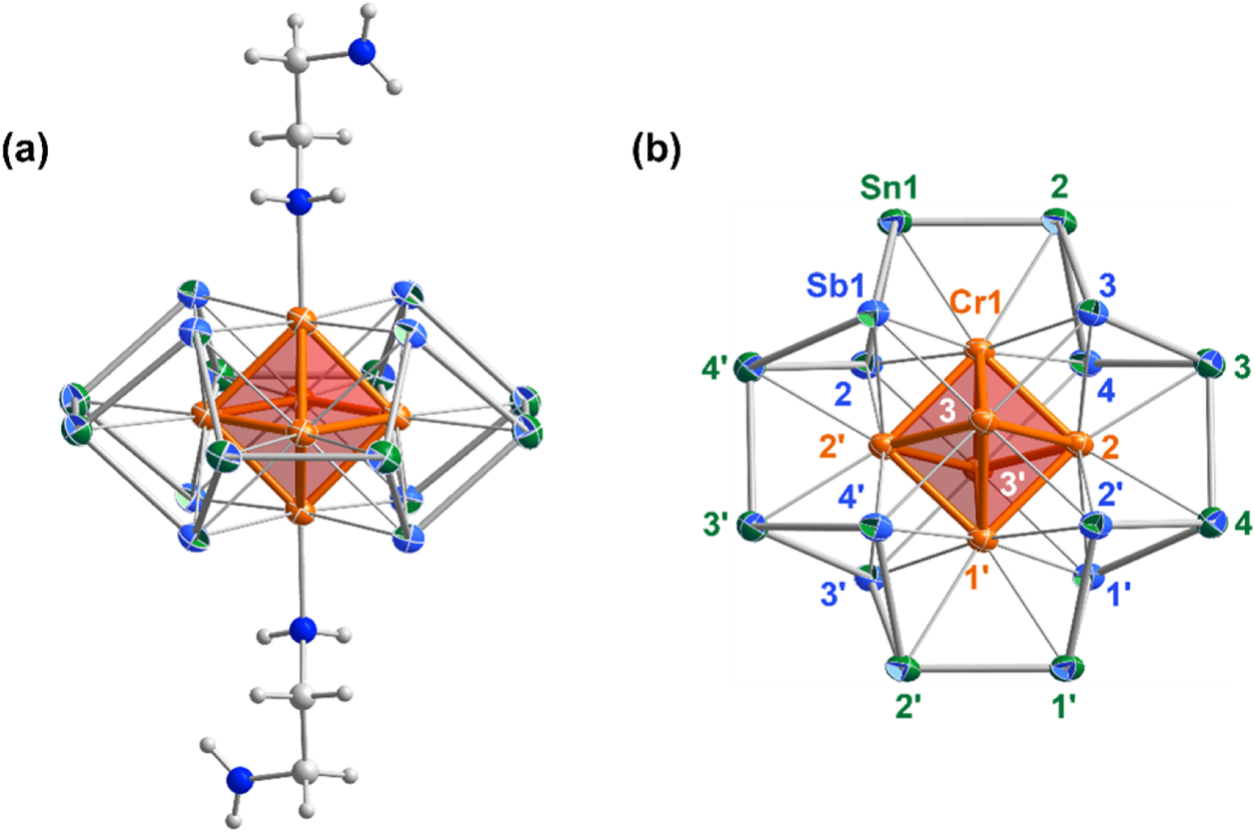
Molecular structure of [Cr_6_@Sn_8_Sb_8_(en)_2_]^3–^ with 50% probability ellipsoids, viewed (a) perpendicular to and (b) along the approximate 4-fold rotational axis. As Sn and Sb cannot be distinguished by X-ray crystallography, these atoms are displayed as two-color octants, with the outer color representing the more likely atom type at each atomic position based on our density functional theory (DFT) calculations (*vide infra*). The two en ligands are omitted from the second view for clarity.

**Figure 3:**
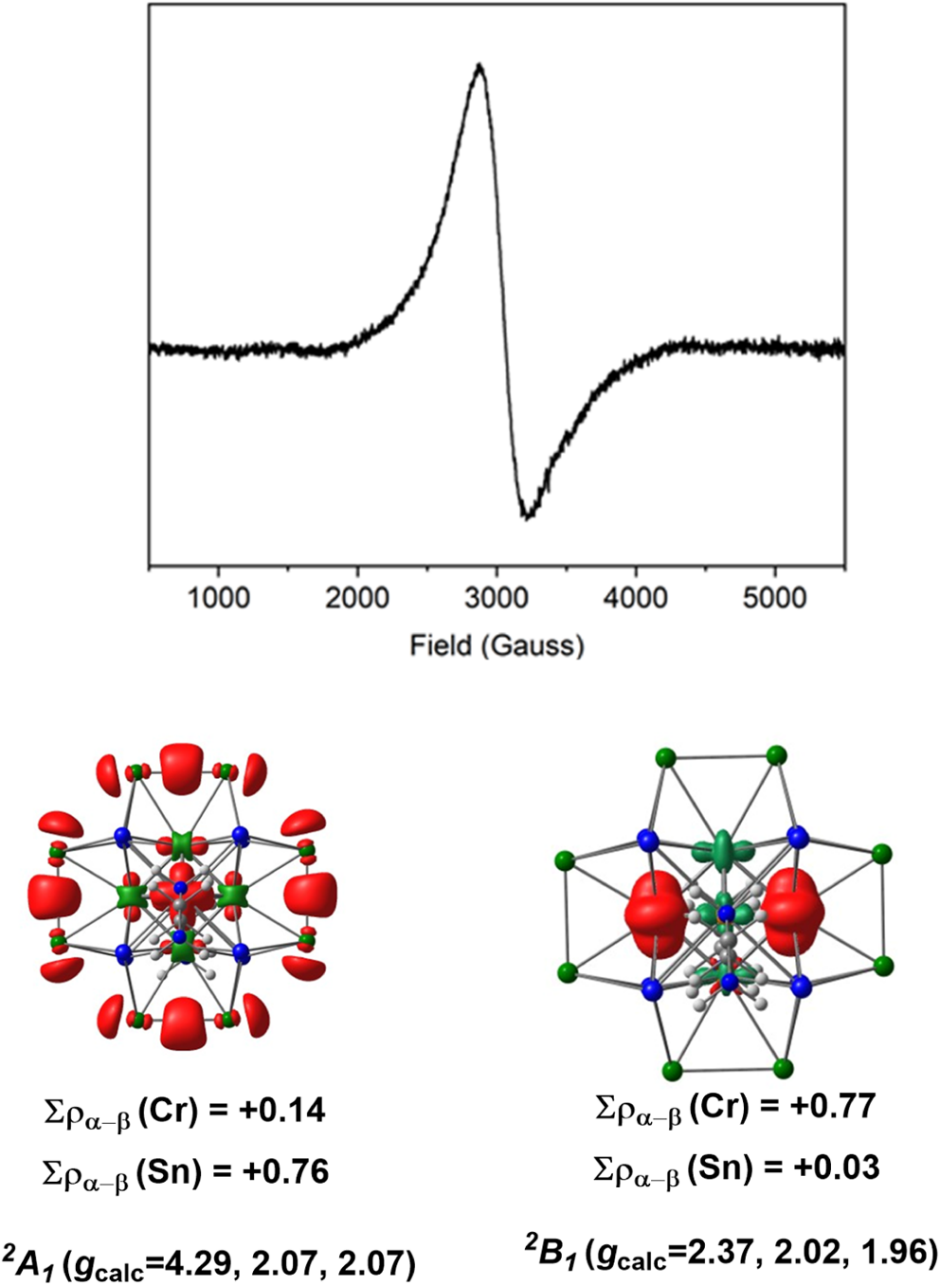
X-band (9.3943 GHz) EPR spectrum of a crystalline sample of **1** in DMF and net spin densities for the ^2^A_1_ (“Sn radical”) and ^2^B_1_ (“Cr_6_ radical”) states of [Cr_6_@Sn_8_Sb_8_(en)_2_]^3–^. Contour values for the isosurface plots are 0.001 e/au^3^.

**Table 1:** Summary of X-ray Data and Relative Energies (eV) and Selected Bond Lengths (All in Å) for the ^2^A_1_ and ^2^B_1_ States of [Cr_6_@Sn_8_Sb_8_(en)_2_]^3–^ ^a^

		E/eV	Cr_eq_–Cr_eq_	Cr_eq_–Cr_ax_	Cr_eq_–Sb	Cr_ax_–Sb	Cr_eq_–Sn	Sn–Sn	Sn–Sb	references
X-ray			2.453(2)–2.457(1)	2.480(2)–2.482(2)	2.739(2)–2.754(2)	2.778(1)–2.790(1)	2.787(1)–2.809(2)	2.9353(9)–2.9293(7)	2.9106(8)–2.9262(8)	this work
DFT	^2^A_1_ (Sn radical)	0.00	2.39	2.42	2.78–2.79	2.78–2.80	2.89–2.91	3.04–3.05	2.97–2.98	
	^2^B_1_ (Cr_6_ radical)	+0.03	2.44–2.45	2.36–2.47	2.78–2.80	2.81–2.85	2.85–2.93	2.96–2.99	2.96–2.99	

	Cr_6_E_8_(PEt_3_)_6_		Cr–Cr		Cr–E		Cr–P			
	Cr_6_S_8_(PEt_3_)_6_		2.59–2.60		2.33–2.35		2.39–2.42			^26^
	Cr_6_Se_8_(PEt_3_)_6_		2.67–2.71		2.43–2.45		2.41–2.43			^26^
	Cr_6_Te_8_(PEt_3_)_6_		2.82–2.86		2.65–2.67		2.43–2.45			^26^

a A structural comparison to members of the Cr_6_E_8_(PR_3_)_6_ family is shown in the lower half of the table.

It is important to acknowledge that we are not able to distinguish Sn from Sb in the diffraction data because their atomic numbers differ by only one – this is a common problem in Zintl cluster chemistry where elements from groups 14 and 15 of the same period are present.^28,29^ However, an 8:8 Sn/Sb ratio in the product is supported by energy dispersive X-ray (EDX) analysis (Supporting Information, Figure S4), where the measured ratio is 7.9:8.4. From this data alone, we cannot absolutely exclude an alternative 7:9 formulation, but given that the X-ray structure places the period 5 elements (Sn/Sb) in two quite different crystallographic positions in an 8:8 ratio, this seems the most likely formulation. ESI mass spectrometry performed on freshly prepared samples of **1** in DMF solution (Supporting Information, Figures S5 and S6) shows evidence for extensive fragmentation, as is commonly observed in Zintl cluster chemistry. There is, however, a small peak at *m*/*z* 1401.8384 that could be assigned to the dianion [{[K­(18-C-6)]_2_Cp}­Sn_8_Sb_8_Cr_4_]^2–^, again consistent with an 8:8 ratio of Sn to Sb. These data are discussed in more detail in the Supporting Information.

In order to establish the precise positions of Sn and Sb within the cluster, we have turned to density functional theory (DFT) to identify the most stable arrangements of atoms. A systematic survey of all possible permutations of Sn and Sb in **1** is beyond the scope of this paper, but we have separately optimized geometries for four distinct models including the one shown in Figure 2 where the Sn atoms lie in the equatorial plane, and the Sb atoms cap the eight triangular faces of the Cr_6_ octahedron. We have also considered an alternative where the positions are reversed (Sb atoms in the equatorial plane and Sn in the face-capping sites), another where half of the Sn and Sb are swapped, and a third where a single Sb is present in the equatorial plane and a single Sn in one of the capping sites. Details are collected in the Supporting Information, Figure S7, but the first of the above arrangements, with all eight Sn atoms in the equatorial plane and all eight Sb in the face-capping positions, proves to be significantly more stable than any of the alternatives, and the match to the experimental bond lengths is also the best. To reflect the ambiguity over the positions of the Sn and Sb atoms, they are displayed as two-color octants in Figure 2, with the outer color representing the atom at that site in the most stable computed structure. With the positions of the Sb and Sn atoms established, we can confirm that the average Cr–Sn (2.798 Å) and Cr–Sb (2.760 Å) bond lengths in **1** are fully consistent with those in known organometallic compounds such as R_3_SnCr­(CO)_3_C_5_Me_5_ (R = Ph, Cy); Cr–Sn = 2.765 Å, (av.)^30^ and (ArSb)­Cr­(CO)_5_; Sb–Cr = 2.748 Å^31^ but marginally shorter than those in Zintl cluster anions such as [Sn_9_Cr­(CO)_3_]^4–^; Cr–Sn = 2.864 Å^32^ and [Sb_7_Cr­(CO)_3_]_^3–^_; Cr–Sb = 2.827 Å.^33^ Sn–Sn bond lengths of 2.93 Å are marginally longer than those in the isolated (Sn_2_Sb_2_)^2–^ anion (2.852–2.884 Å),^29^ but very similar to those in [Pd_3_Sn_8_Bi_6_]^4–^ (2.91 Å).^17^ In the context of the following discussion of the electronic structure, it is also significant that the Cr–Sb bond lengths are much longer than the corresponding Cr–Te bonds in Cr_6_Te_8_(PR_3_)_6_ (ca. 2.64 Å).

### Electronic Structure

Density functional theory has been used to explore the potential energy surface of the cluster anion, [Cr_6_@Sn_8_Sb_8_(en)_2_]^3–^, with the aim of establishing (a) the identity of the electronic ground state and (b) the origin of the very short Cr–Cr bonds. In the X-ray structure, the Cr_6_@Sn_8_Sb_8_ core has approximate *D*_4*h*_ symmetry, but the internal structure of the en ligands precludes the use of such high symmetry in the actual calculations. The conformation of the en ligands has therefore been selected to give the highest possible symmetry, *C*_2*v*_, and all calculations reported here are performed using that point group. All calculations were performed using the Amsterdam Density Functional (ADF) package, version 2024.101;^34^ full details are given in the Experimental Section. Using the Perdew, Burke, and Ernzerhof (PBE) functional,^35^ we have located two quite distinct but closely spaced doublet states, ^2^B_1_ and ^2^A_1_, on the potential surface. Optimized bond lengths of both are compared to the experimental values in Table 1, and net spin densities of the two states are listed in Figure 3. The frontier orbital region of [Cr_6_@Sn_8_Sb_8_(en)_2_]^3–^ is shown in Figure 4. In ^2^B_1_ (referred to henceforth as the “Cr_6_ radical state”), the unpaired electron is in the 3b_1_ orbital (Figure 4) localized on the Cr_6_ core, while in ^2^A_1_ (the “Sn radical state”), it is localized in 5a_1_, a linear combination of Sn–Sn bonding orbitals. Despite the very different localization of the unpaired electron, the Cr_6_ and Sn radical states are very close in energy, the latter being more stable by only 0.03 eV at this level of theory. Given the approximations in our computational model (most notably the absence of K^+^ counterions, the effect of which we model only approximately through the imposition of a continuum with a high dielectric constant), we consider this difference too small to be definitive. We therefore turn to a comparison with the available experimental evidence (both structural and spectroscopic) to identify the ground state. In the Sn radical state (^2^A_1_), the optimized structure of the Cr_6_@Sn_8_Sb_8_ core retains almost perfect *D*_4*h*_ symmetry despite the presence of the en ligands, but the Cr–Cr and Sn–Sn bond lengths are shorter and longer, respectively, than the experimental values (Table 1). The Cr_6_ radical state (^2^B_1_), in contrast, shows more significant deviations from *D*_4*h*_ symmetry with equatorial Cr–Cr bond lengths distributed over a range of ∼0.1 Å. We can trace this distortion to a first-order Jahn–Teller effect: the singly occupied molecular orbital (SOMO) in the ^2^B_1_ state, 3b_1_, correlates with one component of a degenerate pair (the other being 6a_1_) in *D*_4*h*_ symmetry. The ^2^A_1_ state, in contrast, correlates with a nondegenerate ^2^B_1g_ state in the *D*_4*h*_ limit, so the Cr_6_ core is not intrinsically unstable with respect to a first-order distortion. The nature and origin of the Jahn–Teller instability are discussed in more detail in the Supporting Information, Figure S8, but it suffices to note that the average bond lengths for the Cr_6_ radical state offer a closer match to experiment, for both Cr–Cr and Sn–Sn bonds, than those for the Sn radical alternative. The structural differences between the two states are consistent with the transfer of an electron from a Cr–Cr bonding orbital (3b_1_ in Figure 4) to one with a Sn–Sn bonding character (5a_1_). The second piece of experimental evidence that we have at our disposal is the X-band EPR spectrum (measured at 95 K), which shows a broad isotropic signal centered on *g* = 2.21 (Figure 3). The computed *g* values for the ^2^B_1_ state are 1.98, 2.02, and 2.20, the average of which, 2.09, is in reasonable accord with the experimental value (the distribution of values reflecting the distortions from perfect *D*_4*h*_ symmetry noted above). In contrast, the computed *g* tensor for the ^2^A_1_ state is strongly axial, with principal values of 4.00, 2.06, and 2.05. We find no evidence of a signal around *g* = 4 in the spectrum, leading us to conclude that the Cr_6_ radical state, ^2^B_1_, is the more plausible candidate for the ground state despite the fact that it is calculated to be marginally less stable than the Sn radical alternative. We emphasize again that the energetic difference of 0.03 eV between the two states is well below the threshold that we would consider significant, given the approximations in our model.

**Figure 4:**
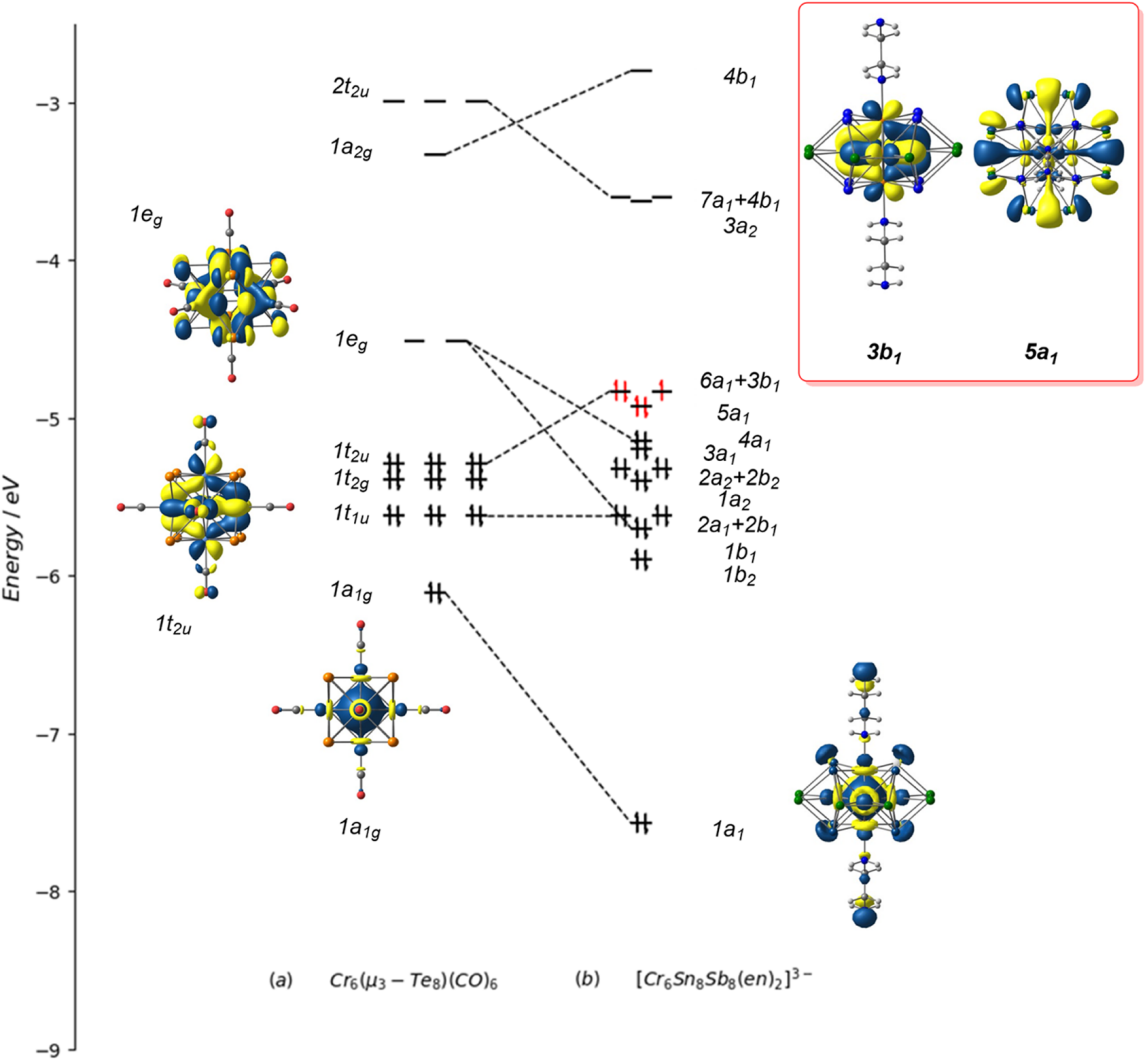
Comparison of the frontier orbitals of (a) Cr_6_Te_8_(CO)_6_ and (b) [Cr_6_@Sn_8_Sb_8_(en)_2_]^3–^. The orbitals of the latter have been grouped to emphasize the degeneracies that are present in the *D*_4*h*_ limit. Contour values for the isosurface plots are 0.025 e^1/2^/au^3/2^.

The valence electron count on the Cr_6_ core is a matter of some importance in the context of the wider comparison with the Cr_6_E_8_(PR_3_)_6_ family, all of which have a count of 20 on the formal Cr^16+^ core. To identify the corresponding count in [Cr_6_@Sn_8_Sb_8_(en)_2_]^3–^, we first establish the charge state of the Sn_8_Sb_8_ ligand. The Sb_12_ unit in [La@Sb_12_]^3–^ is unambiguously Sb_12_^6–^, so each Sb_4_ unit carries a net charge of −2. The Zintl–Klemm pseudoelement concept establishes an isolobal relationship between Sb_4_^2–^ and (Sn_2_Sb_2_)^4–^, which then suggests that the Sn_8_Sb_8_ ligand carries a formal charge of −16. This is the case in the Cr_6_ radical state (^2^B_1_), where we can identify 44 occupied valence orbitals localized on the (Sb_8_Sn_8_)^3–^ unit, up to and including 5a_1_ in Figure 4. Charge balance then requires that the Cr_6_ core carries a charge of +13 and therefore a valence electron count of 23, three electrons more reduced than the formally 20-electron Cr_6_^16+^ cores in Cr_6_E_8_(PR_3_)_6_ and Cr_6_E_8_(CO)_6_. In the Sn radical state (^2^A_1_), in contrast, one electron is transferred from the 5a_1_ orbital to 3b_1_, leaving only 87 electrons on the Sn_8_Sb_8_^15–^ unit and a compensating charge and valence electron count of +12 and 24, respectively, on the Cr_6_ core. For the reasons set out in the previous paragraph, we favor the Cr_6_ radical state as the most likely candidate for the ground state, but in either case, the electron count at the Cr_6_ core is significantly higher than the characteristic value of 20 for Cr_6_E_8_(PR_3_)_6_.

Cotton and Haas first set out the basic molecular orbital framework of octahedral clusters in 1964,^36^ and the Kohn–Sham molecular orbital array for a typical member of this family, Cr_6_Te_8_(CO)_6_ (formally Cr_6_^16+^), is also shown in Figure 4. The 12 Cr–Cr bonding orbitals of the octahedron (one per edge) transform as a_1g_ + e_g_ + t_1u_ + t_2u_ + t_2g_, all of which are occupied apart from 1e_g_, giving the total valence electron count of 20 noted above. In [Mo_6_Cl_14_]^2–^, in contrast, the 1e_g_ orbital is also occupied, raising the count to 24, equating to a single 2-center-2-electron Mo–Mo bond per edge. The 1e_g_ orbital (Figure 4) is simultaneously M–M bonding and M–E antibonding (π*), so its occupation will strengthen M–M bonding at the expense of ME bonding. Clearly, Mo–Mo bonding dominates in [Mo_6_Cl_14_]^2–^ and other clusters of the heavier transition metals, where counts of 24 prevail, while M–E bonding is relatively more significant in the Cr_6_E_8_L_6_ family, where counts of 20 are the norm. The most striking feature of the comparison of Cr_6_E_8_(CO)_6_ with [Cr_6_@Sn_8_Sb_8_(en)_2_]^3–^ shown in Figure 4 is that the 1e_g_ orbital is substantially stabilized (and split) in the latter, to the extent that the stable electron count is 23 rather than 20: Cr-ligand π bonding is clearly rather weaker than that in typical Cr_6_E_8_L_6_ clusters, while Cr–Cr bonding is rather stronger. The weak Cr–Sb π bonding is reflected in the long Cr–Sb bonds (2.74–2.79 Å) compared to 2.65–2.67 Å for the Cr–Te bonds in Cr_6_Te_8_(PEt_3_)_6_.^37^ We can trace the origin of the weak π bonding to the cyclic structure of the Zintl-ion ligand, which ties up precisely half of the 16 orbitals that would be available for π donation in the E_8_ array in the Sn–Sb σ bonds.

## Conclusions

In this paper, we have reported the synthesis of a new cluster, [Cr_6_@Sn_8_Sb_8_(en)_2_]^3–^, containing an octahedral Cr_6_ core encapsulated within a Sn_8_Sb_8_ Zintl-ion ligand. The remarkably short Cr–Cr bonds in the Cr_6_ core mark this cluster out as being electronically quite distinct from members of the Cr_6_E_8_(PEt_3_)_6_ family, where the Cr–Cr bond lengths are up to 0.3 Å longer. The stronger Cr–Cr bonding stems from a valence electron count of 23, three more than the typical count for the phosphine-terminated clusters, and in that sense, the cluster bears a closer resemblance to the heavier Mo_6_, W_6_, and Re_6_ analogues where electron counts of 24 are typical. The fundamental differences in electron count can be traced to the rather unusual properties of the face-capping Sb atoms of the Sn_8_Sb_8_ “ligand”, which have a reduced capacity to act as π donors compared to an array of 8 isolated chalcogenide ions.

## Experimental Section

### Synthesis

All manipulations and reactions were performed under a nitrogen atmosphere by using standard Schlenk or glovebox techniques. Ethylenediamine (Aldrich, 99%) and toluene (Aldrich, 99.8%) were freshly distilled from sodium/benzophenone. DMF (Aldrich, 99.8%) was distilled from CaH_2_ under N_2_ and stored under nitrogen prior to use. 18-crown-6 (1,4,7,10,13,16-Hexaoxacyclooctadecane, 98%) purchased from Sigma-Aldrich was dried in vacuum for 1 day prior to use. The precursor K_2_SnSb was synthesized by fusing a mixture of the elements in a 2:1:1 ratio in a Nb tube at 900 °C for 2 days.^38^ CrCp_2_ was synthesized according to the reported literature preparation.^39^

#### Synthesis of {[K­(18-C-6)]_2_Cp}_3_{[Cr_6_@Sn_8_Sb_8_(en)_2_]}, **1**

K_2_SnSb (90 mg, 0.282 mmol) and 18-crown-6 (115 mg, 0.435 mmol) were dissolved in 2.5 mL of en and stirred at 50 °C for 0.5 h to yield a brown solution. CrCp_2_ (26 mg, 0.143 mmol) was added slowly over 0.5 h, and then the mixture was stirred vigorously at room temperature for 3 h. The resulting brown solution was centrifuged and filtered with standard glass wool, then carefully layered with 3 mL of toluene. After 2 weeks, long black plate-like crystals of **1** formed in the test tube with approximately 21% yield (based on the amount of K_2_SnSb used). The addition of PPh_3_ does not alter the yield of **1** or lead to the formation of clusters in more oxidized states.^40^

### Structural Characterization

Suitable single crystals of **1** were selected for X-ray diffraction analyses. Crystallographic data were collected on a Rigaku XtalAB Pro MM007 DW diffractometer with graphite monochromated Cu Kα radiation (λ = 1.54184 Å). Structures were solved using direct methods and then refined using SHELXL-2014 and Olex2 to convergence, in which all of the nonhydrogen atoms were refined anisotropically during the final cycles. All hydrogen atoms of the organic molecule were placed by geometrical considerations and were added to the structure factor calculation. Both compounds are air- and moisture-sensitive in solution and in the solid state. Crystallographic data is deposited in the Cambridge Crystallographic Database (CCDC), entry 2212805.

### Electrospray Ionization Mass Spectrometry (ESI-MS)

Electrospray ionization mass spectrometry (ESI-MS) was performed in negative-ion mode on an LTQ linear ion trap spectrometer by an Agilent Technologies ESI-TOF-MS(6230). The spray voltage was 5.48 kV, and the capillary temperature was kept at 300 °C. The capillary voltage was 30 V, and the sheath gas was maintained at 50 °C. The samples were made up inside a glovebox under an N_2_ atmosphere and rapidly transferred to the spectrometer in an airtight syringe by direct infusion with a Harvard syringe pump at 0.2 mL/min.

### Energy Dispersive X-ray (EDX) Spectroscopy

EDX analyses were performed by using a scanning electron microscope (FE-SEM, JEOL JSM-7800F, Japan). Data acquisition was performed with an acceleration voltage of 15 kV and an accumulation time of 60 s.

### Electron Paramagnetic Resonance Spectroscopy

The EPR spectrum of a crystalline sample of **1** was collected at 94 K in the *Sharing of Apparatus Management Platform* at Nankai University. X-band measurements were performed with a Bruker-E580-10/12 spectrometer equipped with an FT/CW microwave bridge, a Flexline resonator, and an ESR-900 liquid helium cryostat. Nonsaturating conditions were found, with a microwave frequency of 9.3943 GHz, a microwave power of 47.43 mW, a modulation amplitude of 0.2 mT, a time constant of 50.00 ms, and a sweep rate of 300 s over 100 mT.

### Computational Methods

All calculations described in this paper were performed with the Amsterdam Density Functional (ADF) package of program, version 2024.101.^34^ The exchange-correlation functional proposed by Perdew, Burke, and Ernzerhof (PBE) was used throughout,^35^ and scalar relativistic effects were introduced using the zeroth-order regular approximation (ZORA).^41^ For all geometry optimizations, a triple-ζ quality basis set of Slater-type orbitals was used,^42^ supplemented by two sets of polarization functions. Electrons up to and including 2p (Cr), 4p (Sn, Sb), and 1s (C, N, F) were treated using the frozen core approximation. The conductor-like screening model (COSMO) was applied to simulate the confined crystal environment in the experiment with a dielectric constant of 100.^43^ The nature of the stationary points was determined by calculating the frequencies in the harmonic approximation. In the case of the [Cr_6_@Sn_8_Sb_8_(en)_2_]^3–^ system, the analysis was complicated by the presence of the en ligands, which contain several single bonds about which rotation is almost barrierless. In the chosen conformation (with *C*_2*v*_ point symmetry), the ^2^A_1_ state has two residual imaginary frequencies (60.5 and 55.4 *i*cm^–1^) associated with torsions about the Cr–N bonds. The marginally less stable ^2^B_1_ state also has two imaginary frequencies with similar characteritics at 61.1 and 56.3 *i*cm^–1^, along with a third imaginary mode (649.8 *i*cm^–1^). Attempts to eliminate this imaginary mode by removing all symmetry elements led to convergence on the more stable ^2^A_1_ state, which necessarily transforms as the same representation in symmetries lower than *C*_2*v*_.

## Supplementary Material



## Notes

Characterization data for **1**. Further details of computational analysis and optimized coordinates for all calculated species (PDF)
