# Supplementary material for: Synthesis, Structure, and Electronic Properties of [Cr6@Sn8Sb8(en)2]3–: A Cr6 Octahedron Encapsulated in a Zintl-Ion Ligand
Source: J Am Chem Soc. 2025 Jul 8;147(28):24309–16. doi: 10.1021/jacs.5c00515 (PMC12272683; doi:10.1021/jacs.5c00515)
Supplement: Supplementary file 1 [file ja5c00515_si_001.pdf]

Supplementary Information

Synthesis, structure and electronic properties of $[Cr_6@Sn_8Sb_8(en)_2]^{3-}$: a Cr_6 octahedron encapsulated in a Zintl-ion ligand

Wei-Xing Chen,[†]a Aidan Manley,[‡]a Zi-Sheng Li,[‡] John E. McGrady,*[‡] and Zhong-Ming Sun*[†]

[†] State Key Laboratory and institute of Elemento-Organic Chemistry, Tianjin Key Lab for Rare Earth Materials and Applications, School of Materials Science and Engineering, Nankai University, Tianjin 300350, China
[‡] Department of Chemistry, University of Oxford, South Parks Road, Oxford OX1 3QR, UK

a These authors contributed equally

Table of Contents

Section 1: Crystallographic Supplementary Information..2
Section 2: ESI-MS Studies...4
Section 3: Energy Dispersive X-ray (EDX) Spectroscopic Analysis..................................5
Section 4: Quantum Chemical Calculations...6
- **4.1 Analysis of impact of en/F$^-$ substitution.**
- **4.2 Models of Sn/Sb disorder**
- **4.4 Analysis of the Jahn-Teller distortions in the ^2E_u state**
- **4.5 Condensed summary of output files, including optimized cartesian coordinates**

Section 5: Supplementary References...38

Section 1: Crystallographic Supplementary Information.

Suitable single crystals of **1** were selected for X-ray diffraction analyses. Crystallographic data were collected on Rigaku XtalAB Pro MM007 DW diffractometer with graphite monochromated Cu Kα radiation (λ = 1.54184 Å). The crystal structure was solved using direct methods and then refined using SHELXL-2014 and Olex2.[1] All the non-hydrogen atoms were refined anisotropically, except for those in split positions. The uncoordinated solvent molecules in **1** could not be modeled properly, so are removed using SQUEEZE in PLATON.[2] A summary of the crystallographic data for **1** is listed in Table S1. CCDC entry 2212805 contains the supplementary crystallographic data for **1**. These data are provided free of charge by the Cambridge Crystallographic Data Centre.

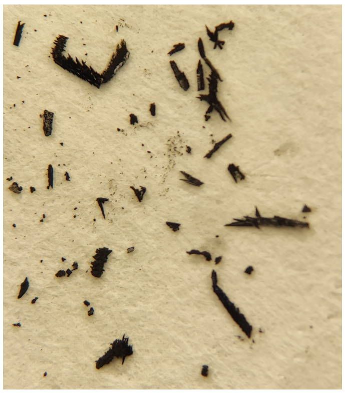

Figure S1. Crystals of **1**.

Table S1. X-ray measurements and structure solutions.

Compound	**1**
Empirical formula	$C_{92}H_{176}Cr_6K_6N_4O_{36}Sb_8Sn_8$
Formula weight	4384.48
Temperature/K	100.00(10)
Crystal system	triclinic
Space group	$P\bar{1}$
a/Å	16.6791(3)
b/Å	17.6100(4)
c/Å	17.8033(4)
α/°	107.052(2)
β/°	103.040(2)
γ/°	115.882(2)
Volume/Å^3	4100.10(17)
Z	1
ρ_{calc} g/cm^3	1.776
$\mu(Cu_{K\alpha})$/mm^{-1}	24.707
F(000)	2110.0
2Θ range /°	8.256 to 133.994
Reflections collected	41273
Data/restraints/parameters	14565/17/728

GooF (all data)[b]	1.060
R_1/wR_2 (I>=2σ (I))[a]	R_1 = 0.0614, wR_2 = 0.1662
R_1/wR_2 (all data)	R_1 = 0.0707, wR_2 = 0.1753
Max. peak/hole /$e^{-}\cdot Å^{-3}$	2.91/-1.97
CCDC	2212805

[a] $R_1 = \sum||F_o|-|F_c||/\sum|F_o|$; $wR_2 = \{\sum w[(F_o)^2-(F_c)^2]^2/\sum w[(F_o)^2]^2\}^{1/2}$

[b] $GooF = \{\sum w[(F_o)^2-(F_c)^2]^2/(n-p)\}^{1/2}$

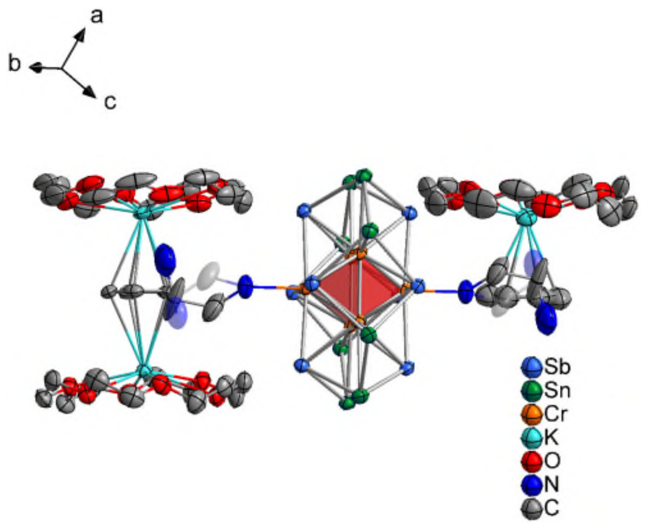

Figure S2. Asymmetric unit of **1**. Thermal ellipsoids are drawn at 50% probability. Positions of disordered atoms are given in semi-transparent mode. H atoms are omitted for clarity. Sn and Sb atoms cannot be distinguished by X-ray crystallography, so the colors of the Sn (green) and Sb (blue) atoms reflect the most likely positions based on the DFT calculations.

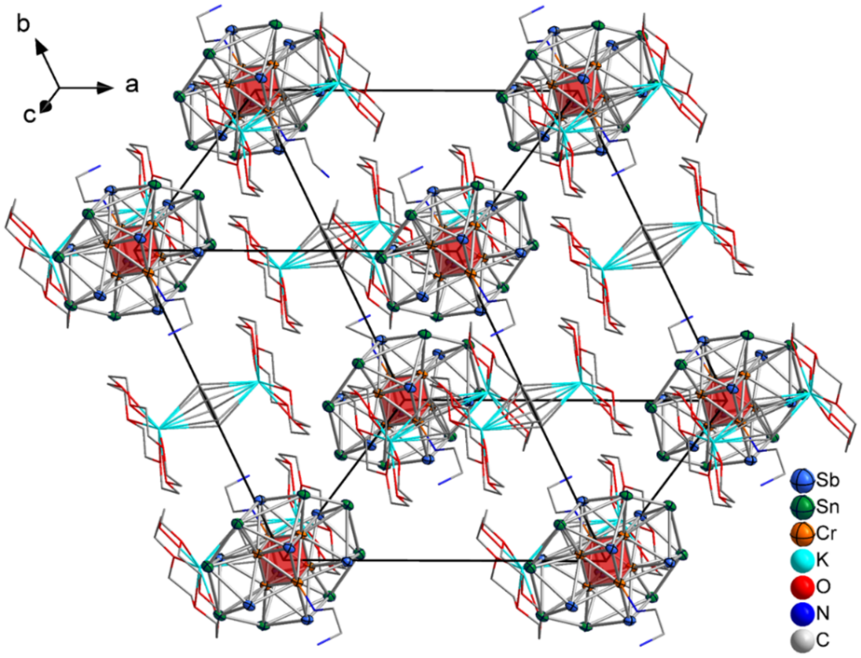

Figure S3. Unit cell of **1**. Minor components and disorder atoms in the cluster site are omitted for clarity. Sn and Sb atoms cannot be distinguished by X-ray crystallography, so the colors of the Sn (green) and Sb (blue) atoms reflect the most likely positions based on the DFT calculations.

Section 2: Energy Dispersive X-ray (EDX) Spectroscopic Analysis

The results of EDX analysis on complexes **1** are presented in Figure S4 and Table S2. EDX analysis were performed by a scanning electron microscope (FE-SEM, JEOL JSM-7800F, Japan). Data acquisition was performed with an acceleration voltage of 15 kV and an accumulation time of 60 s. The deviation in the quantity of K from the expected value may be due to the irregular surface of a crystals after exposure to air.

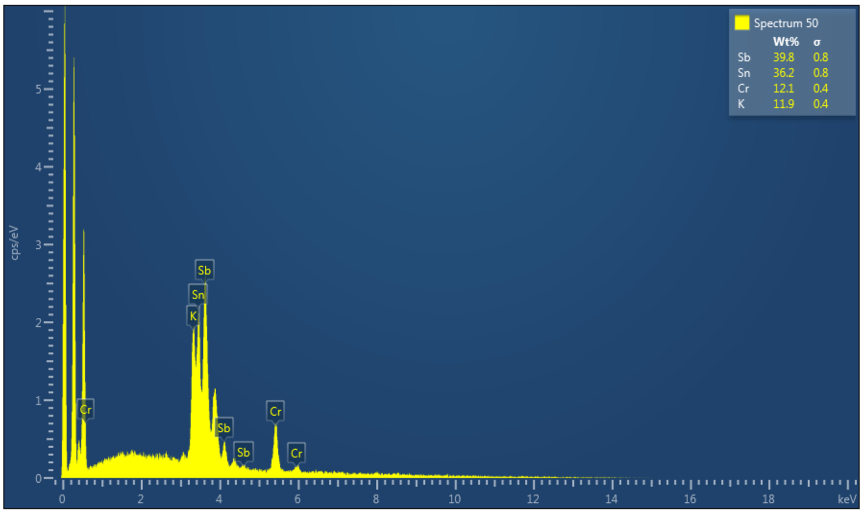

Figure S4. EDX of complex **1**.

Table S2. EDX analysis of complex **1**.

Element	wt%	σ	Experimental / Calculated Atom
K	11.9	0.4	7.8/6.0
Cr	12.1	0.4	6.0/6.0
Sn	36.2	0.8	7.9/8.0
Sb	39.8	0.8	8.4/8.0

Section 3: ESI-MS Studies

Negative ion mode ESI-MS of a freshly-prepared DMF solution of crystals of **1** was measured on an LTQ linear ion trap spectrometer by Agilent Technologies ESI-TOF-MS (6230). The spray voltage was 5.48 kV and the capillary temperature was kept at 300 °C. The capillary voltage was 30 V. The samples were prepared inside a glovebox and rapidly transferred to the spectrometer in an airtight syringe by direct infusion with a Harvard syringe pump at 0.2 mL/ min. The cluster undergoes extensive fragmentation under ESI-MS conditions, an observation that is common in Zintl cluster chemistry, but a peak at m/z = 1401.8384 can be assigned to the dianion $[[K(18\text{-}C\text{-}6)]_2CpCr_4Sn_8Sb_8Cr_4]^{2-}$ (Figures S5, S6). The $[[K(18\text{-}C\text{-}6)]_2Cp]^+$ unit is a component of the crystal structure of **1**, so its detection in the ESI-MS experiment, presumably as a cation bound to a $[Cr_4Sn_8Sb_8]^{3-}$ anion through electrostatic forces, is unsurprising. This assignment suggests that the two axial Cr(en) units are readily lost from **1** as neutral fragments.

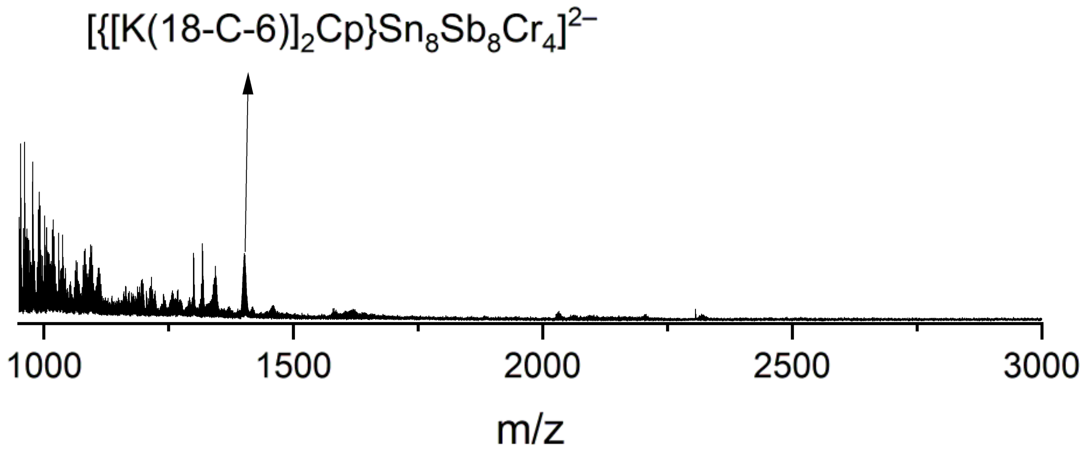

Figure S5. Overview ESI mass spectrum in negative ion mode of a freshly dissolved crystalline sample of **1** in DMF.

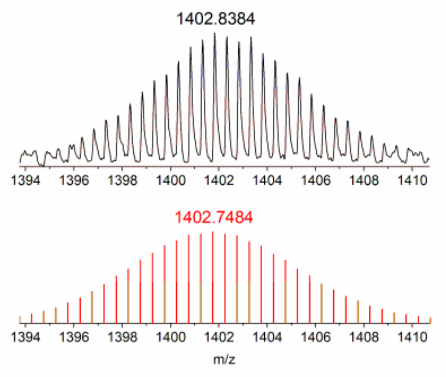

Figure S6. Measured (black) and simulated (red) spectrum of the fragment $[\{[K(18\text{-}C\text{-}6)]_2Cp\}Sn_8Sb_8Cr_4]^{2-}$.

Section 4: Quantum Chemical Calculations

4.1 Comparison of results with en and with F^- model ligands.

In the following section, we use $F^–$ as a model ligand to replace en. The reason for this choice is that the monoatomic ligand allows us to use the full D_{4h} point symmetry of the $Cr_6Sn_8Sb_8$ core in the calculations, which in turn leads to a rigorous symmetry-based distinction between the ^2E_u and $^2B_{1g}$ states. In contrast, these states have the same symmetry in the lower symmetry imposed by the internal structure of the en of NH_3 ligands, and this leads to significant SCF convergence issues. To test the impact of replacing en with $F^–$, we report below in Table S3 the optimized energies and geometries of both states with both en and $F^–$. Structurally, there is very little difference between the states with the two ligands: Cr-Cr distances differ by at most 0.02 Å. Energetically, the $F^–$ ligand stabilizes the ^2E_u state by 0.24 eV compared to the en case. This difference is a result of the π-donor character of the $F^–$ ligand, which is more significant in the ^2E_u state where there is a singly-occupied orbital localized on the Cr_6 core.

Table S3 Comparison of optimized structures for model systems with the full en ligand (top) and the $F^–$ model ligand (bottom).

		E/eV	Cr_{eq}–Cr_{eq}	Cr_{eq}–Cr_{ax}	Cr_{eq}–Sb	Cr_{ax}–Sb	Cr_{eq}–Sn	Sn–Sn	Sn–Sb
$[Cr_6Sn_8Sb_8(en)_2]^{3–}$			2.453(2)-2.457(1)	2.480(2)-2.482(2)	2.739(2)-2.754(2)	2.778(1)-2.790(1)	2.787(1)-2.809(2)	2.9353(9)-2.9293(7)	2.9106(8)-2.9262(8)
DFT: $[Cr_6Sn_8Sb_8(en)_2]^{3–}$	$^2B_{1g}$	0.00	2.39	2.42	2.78-2.79	2.78-2.80	2.89-2.91	3.04-3.05	2.97-2.98
	$^2E_u{}^†$	+0.03	2.44-2.45	2.36-2.47	2.78-2.80	2.81-2.85	2.85-2.93	2.96-2.99	2.96-2.99
DFT: $[Cr_6Sn_8Sb_8F_2]^{5–}$	$^2B_{1g}$	0.00	2.39	2.40	2.82	2.80	2.91	3.07	2.97
	$^2E_u{}^†$	-0.21	2.42-2.47	2.37-2.48	2.78-2.81	2.82-2.84	2.85-2.92	2.99-3.02	2.96-2.99

4.2 Models of Sn/Sb disorder in $[Cr_6Sn_8Sb_8F_2]^{5-}$.

In order to establish the most favorable distribution of Sn and Sb across the 16 sites of the Sn_8Sb_8 ring, we have conducted a series of calculations on different disorder models, Figure S7 and Table S4. In these calculations we have replaced the en ligands with the monoatomic F^- in order to maximise the symmetry. **Model 1** corresponds exactly to the ^2E_u ground state reported in the main text, with all Sn atoms in the equatorial positions (E_{eq}), all Sb in the face capping positions (E_{fac}). **Model 2** has the positions of Sn and Sb reversed. In **model 3**, half of the Sn and Sb atoms are swapped to give a C_2-symmetric arrangement. In **model 4**, one Sn and one Sb atom are swapped. The calculations indicate that **model 1** is the most stable, 0.91 eV below **model 2**, 1.80 eV below **model 3** and and 0.36 below **model 4**.

Figure S7 Disorder models for the Sn/Sb atoms.

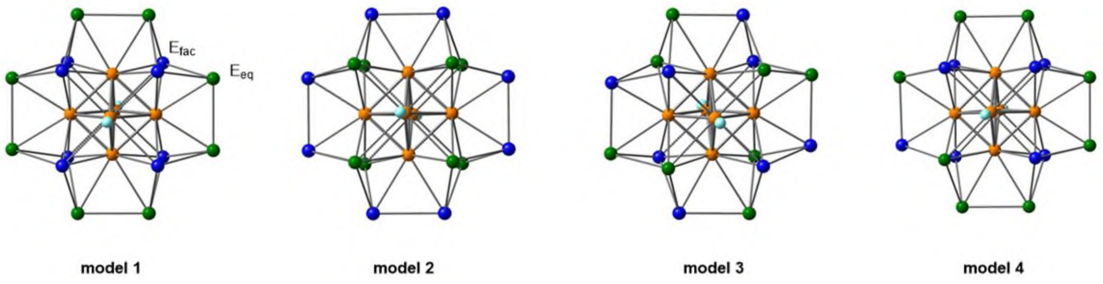

Table S4 Total energies and optimized structural parameters of various models of Sn/Sb disorder. Note that these calculations were done using the F^- model ligand in place of en.

		E/eV	Cr_{eq}–Cr_{eq}	Cr_{eq}–Cr_{ax}	Cr_{eq}–E_{fac}	Cr_{ax}–E_{fac}	Cr_{eq}–E_{eq}	E_{eq}–E_{eq}	E_{eq}–E_{fac}
$[Cr_6Sn_8Sb_8(en)_2]^{3-}$ X-ray			2.453(2)-2.457(1)	2.480(2)-2.482(2)	2.739(2)-2.754(2)	2.778(1)-2.790(1)	2.787(1)-2.809(2)	2.9353(9)-2.9293(7)	2.9106(8)-2.9262(8)
DFT: $[Cr_6Sn_8Sb_8F_2]^{5-}$	**Model 1 (^2E_u)**	0.0	2.42-2.47	2.37-2.48	2.78-2.81	2.82-2.84	2.85-2.92	2.99-3.02	2.96-2.99
	**Model 2**	+0.91	2.45-2.46	2.32-2.39	2.78-2.81	2.82-2.84	2.85-2.92	2.99-3.02	2.96-2.99
	**Model 3**	+1.80	2.42-2.45	2.35-2.43	2.76-2.92	2.82-2.88	2.76-2.92	2.94-2.96	2.95-3.00
	**Model 4**	+0.36	2.38-2.50	2.35-2.43	2.78-2.89	2.81-2.92	2.76-2.94	2.96-3.00	2.96-3.02

4.3 Detailed analysis of the Jahn-Teller distortions of the ^2E_u state of $[Cr_6Sn_8Sb_8F_2]^{5-}$.

We noted in the main text that the ^2E_u state of $[Cr_6Sn_8Sb_8(en)_2]^{3-}$ was necessarily unstable with respect to a distortion that removes the degeneracy of the state. We explore in more detail the nature of this distortion here using the $[Cr_6Sn_8Sb_8F_2]^{5-}$ which allows us to impose strict D_{4h} symmetry, and then explore the consequences of relaxing this constraint. Indeed, we find that in D_{4h} point symmetry, the ^2E_u state is a saddle point on the potential surface with three imaginary frequencies, 176i cm^{-1} (E_u) and 216i cm^{-1} (B_{1g}). Following one of the components of the E_u mode leads to a D_{2h}-symmetric $^2B_{3u}$ state where the equatorial Cr_4 unit is distorted from a square to a rhombus (Figure S9). This is a classic first-order Jahn-Teller distortion which lifts the degeneracy of the $2e_u$ orbital, splitting it into b_{2u} and b_{3u} components, and the net result is a stabilisation of 0.31 eV. The $^2B_{3u}$ state shows one residual imaginary frequency (182i cm^{-1}), and following this leads to the C_{2v}-symmetric $^2A_1$ ground state which is a true minimum with no imaginary frequencies, where the capping CrF units move slightly off the centre of the rhombus. This final distortion from D_{2h} to C_{2v} is relatively minor in energetic terms (it yields a further stabilisation of *ca* 0.04 eV). Overall, then, whilst the low-symmetry distortions connecting the ^2E_u state to $^2A_1$ lead to an overall stabilisation of only 0.35 eV, they are critical in determining the identity of the ground state in the case where there are two or more closely spaced states.

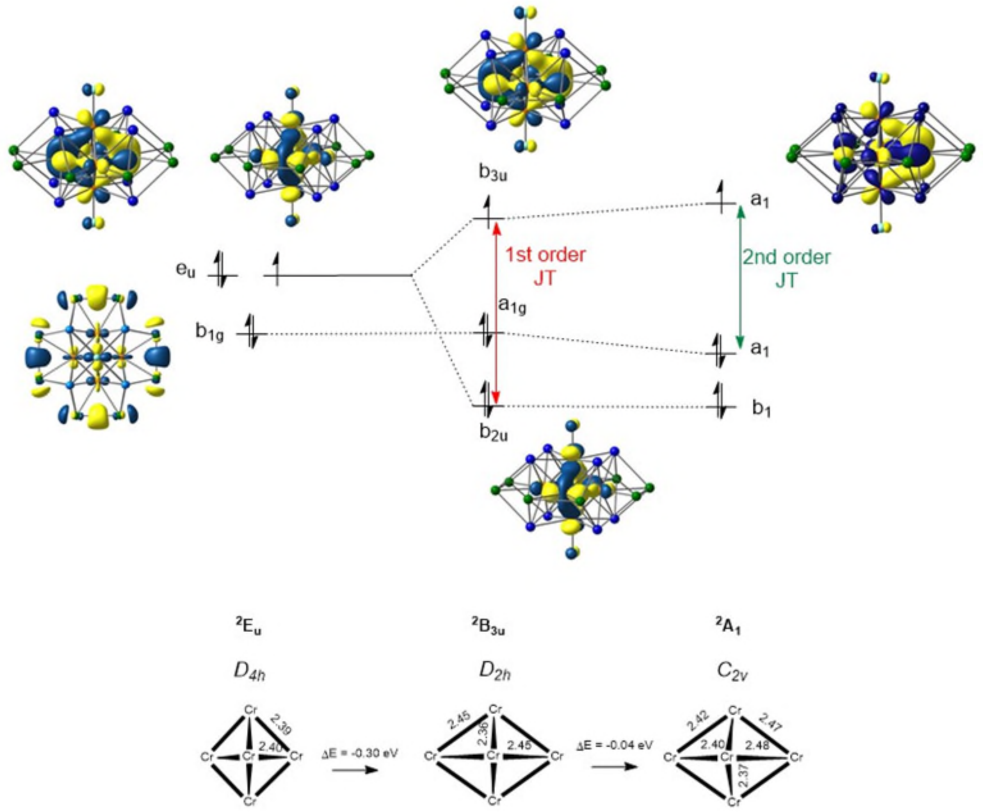

Figure S8. 1st and 2nd order Jahn-Teller instabilities driving the low-symmetry distortion of the Cr_6 core. Contour values for the isosurface plots are 0.025 $e^{1/2}au^{3/2}$.

4.4 Summary details of calculations.

The entries below summarize the key details of the calculations performed, including the energies and optimized cartesian coordinates, but also details of computational set up to allow for reproduction of the results.

(a) $[Cr_6Sn_8Sb_8(en)_2]^{3-}$ ($^2B_{1g}$ state),
(b) $[Cr_6Sn_8Sb_8(en)_2]^{3-}$ (^2E_u state)
(c) $[Cr_6Sn_8Sb_8F_2]^{5-}$ ($^2B_{1g}$ state),
(d) $[Cr_6Sn_8Sb_8F_2]^{5-}$ (^2E_u state),
(e) $Cr_6(\mu_3\text{-Te})_8(CO)_6$.
(f) Disorder model 2 $[Cr_6Sn_8Sb_8F_2]^{5-}$
(g) Disorder model 3 $[Cr_6Sn_8Sb_8F_2]^{5-}$
(h) Disorder model 4 $[Cr_6Sn_8Sb_8F_2]^{5-}$

(a) Title: **cr6sn8sb8en2 $^2B_{1g}$**

Molecular Formula: C4H16Cr6N4Sb8Sn8 Charge: -3.0
Job Type: Geometry Optimization

no of valence electrons: 371.0
spin polarization (alpha-beta = Ms): 1.0

basis set: TZP core: small

frozen cores:
Cr 1s 2s 2p
Sb 1s 2s 2p 3s 3p 3d 4s 4p
Sn 1s 2s 2p 3s 3p 3d 4s 4p
N 1s
C 1s

no of basis functions: 856

functional: PBE

relativistic options: scalar ZORA,MAPA

no symmetry
SCF Energy:
-9.318116 au
-253.5588 eV

-5847.21 kcal/mol
-24464.71 kJ/mol

Technical details of geometry convergence
Optimization method Quasi Newton
required 30 steps to reach convergence

Optimized cartesian coordinates in Angstrom:

0 Sn	1.5211	-0.0000	3.9497
1 Sn	-1.5211	0.0000	3.9497
2 Sn	-4.1740	0.0000	1.2953
3 Sn	-4.1538	0.0000	-1.7501
4 Sn	-1.5265	0.0000	-4.3680
5 Sn	1.5265	-0.0000	-4.3680
6 Sn	4.1538	-0.0000	-1.7501
7 Sn	4.1740	-0.0000	1.2953
8 Sb	1.9732	1.9493	1.7509
9 Sb	1.9732	-1.9493	1.7509
10 Sb	-1.9732	1.9493	1.7509
11 Sb	-1.9732	-1.9493	1.7509
12 Sb	-1.9639	-1.9654	-2.1737
13 Sb	-1.9639	1.9654	-2.1737
14 Sb	1.9639	-1.9654	-2.1737
15 Sb	1.9639	1.9654	-2.1737
16 N	-0.0000	-4.0227	-0.1340
17 N	0.0000	4.0227	-0.1340
18 N	0.0000	7.4205	-1.9272
19 N	-0.0000	-7.4205	-1.9272
20 H	-0.8858	-4.7776	-1.8828
21 H	0.8858	-4.7776	-1.8828
22 H	0.8858	4.7776	-1.8828
23 H	-0.8858	4.7776	-1.8828
24 H	-0.8146	-4.2169	0.4624
25 H	0.8146	-4.2169	0.4624
26 H	-0.8146	4.2169	0.4624
27 H	0.8146	4.2169	0.4624
28 H	0.8846	-6.6451	-0.1888
29 H	-0.8846	-6.6451	-0.1888
30 H	0.8846	6.6451	-0.1888
31 H	-0.8846	6.6451	-0.1888
32 H	0.8132	7.2403	-2.5252
33 H	-0.8132	7.2403	-2.5252

34 H	0.8132	-7.2403	-2.5252
35 H	-0.8132	-7.2403	-2.5252
36 Cr	0.0000	1.7306	-0.2264
37 Cr	-0.0000	-1.7306	-0.2264
38 Cr	0.0000	0.0000	1.4650
39 Cr	-1.6899	0.0000	-0.2202
40 Cr	0.0000	0.0000	-1.9082
41 Cr	1.6899	-0.0000	-0.2202
42 C	-0.0000	-4.9837	-1.2622
43 C	0.0000	4.9837	-1.2622
44 C	-0.0000	-6.4507	-0.8171
45 C	0.0000	6.4507	-0.8171

Symmetry C(2V)

Irreducible representations and SCF occupations

A1 61 \ 60
A2 33 \ 33
B1 48 \ 48
B2 44 \ 44
total 371

Spin densities

	Mulliken	mdc m	mdc	mdc q
Cr	0.11	0.10	0.06	0.07
Cr	0.11	0.10	0.06	0.07
Cr	-0.01	0.00	0.00	0.01
Cr	-0.02	-0.00	-0.00	-0.00
Cr	-0.03	-0.01	-0.01	-0.01
Cr	-0.02	-0.00	-0.00	-0.00
Sb	0.01	0.01	0.01	0.01
Sb	0.01	0.01	0.01	0.01
Sb	0.01	0.01	0.01	0.01
Sb	0.01	0.01	0.01	0.01
Sb	0.01	0.01	0.02	0.02
Sb	0.01	0.01	0.02	0.02
Sb	0.01	0.01	0.02	0.02
Sb	0.01	0.01	0.02	0.02
Sn	0.09	0.09	0.09	0.09
Sn	0.09	0.09	0.09	0.09
Sn	0.10	0.09	0.10	0.10

Sn	0.09	0.09	0.09	0.09
Sn	0.10	0.09	0.10	0.10
Sn	0.10	0.09	0.10	0.10
Sn	0.09	0.09	0.09	0.09
Sn	0.10	0.09	0.10	0.10
N	-0.00	-0.00	0.00	0.00
N	-0.00	-0.00	0.00	0.00
C	-0.00	-0.00	-0.00	-0.01
H	-0.00	0.00	0.00	0.00
H	-0.00	0.00	0.00	0.00
C	-0.00	-0.00	-0.00	-0.01
H	-0.00	0.00	0.00	0.00
H	-0.00	0.00	0.00	0.00
H	-0.00	-0.00	0.00	-0.00
H	-0.00	-0.00	0.00	-0.00
H	-0.00	-0.00	0.00	-0.00
H	-0.00	-0.00	0.00	-0.00
C	-0.00	-0.00	-0.00	0.00
H	0.00	0.00	-0.00	-0.00
H	0.00	0.00	-0.00	-0.00
C	-0.00	-0.00	-0.00	0.00
H	0.00	0.00	-0.00	-0.00
H	0.00	0.00	-0.00	-0.00
N	-0.00	-0.00	-0.00	-0.00
H	0.00	0.00	0.00	-0.00
H	0.00	0.00	0.00	-0.00
N	-0.00	-0.00	-0.00	-0.00
H	0.00	0.00	0.00	-0.00
H	0.00	0.00	0.00	-0.00

<S2> = 0.75427 exact <S2> = 0.75

(b) Title: **cr6sn8sb8en2 ^2E_u**

Molecular Formula: C4H16Cr6N4Sb8Sn8 Charge: -3.0
Job Type: Geometry Optimization

no of valence electrons: 371.0
spin polarization (alpha-beta = Ms): 1.0

no of basis functions: 856

basis set: TZP core: small

frozen cores:
Cr 1s 2s 2p
Sb 1s 2s 2p 3s 3p 3d 4s 4p
Sn 1s 2s 2p 3s 3p 3d 4s 4p
N 1s
C 1s

functional: PBE

relativistic options: scalar ZORA,MAPA

no symmetry
SCF Energy:
-9.316561 au
-253.5165 eV
-5846.23 kcal/mol
-24460.63 kJ/mol

Technical details of geometry convergence
Optimization method Quasi Newton
required 34 steps to reach convergence

Optimized cartesian coordinates in Angstrom:

0 Sn	1.4815	0.0000	3.9482
1 Sn	-1.4815	0.0000	3.9482
2 Sn	-4.2345	0.0000	1.2625
3 Sn	-4.2194	0.0000	-1.7289
4 Sn	-1.4825	-0.0000	-4.4065
5 Sn	1.4825	0.0000	-4.4065

6 Sn	4.2194	-0.0000	-1.7289
7 Sn	4.2345	-0.0000	1.2625
8 Sb	2.0005	1.9287	1.7603
9 Sb	2.0005	-1.9287	1.7603
10 Sb	-2.0005	1.9287	1.7603
11 Sb	-2.0005	-1.9287	1.7603
12 Sb	-1.9915	-1.9400	-2.2119
13 Sb	-1.9915	1.9400	-2.2119
14 Sb	1.9915	-1.9400	-2.2119
15 Sb	1.9915	1.9400	-2.2119
16 N	-0.0000	-3.9615	-0.1473
17 N	0.0000	3.9615	-0.1473
18 N	0.0000	7.3857	-1.8854
19 N	-0.0000	-7.3857	-1.8854
20 H	-0.8863	-4.7394	-1.8853
21 H	0.8863	-4.7394	-1.8853
22 H	0.8863	4.7394	-1.8853
23 H	-0.8863	4.7394	-1.8853
24 H	-0.8144	-4.1477	0.4533
25 H	0.8144	-4.1477	0.4533
26 H	-0.8144	4.1477	0.4533
27 H	0.8144	4.1477	0.4533
28 H	0.8843	-6.5795	-0.1612
29 H	-0.8843	-6.5795	-0.1612
30 H	0.8843	6.5795	-0.1612
31 H	-0.8843	6.5795	-0.1612
32 H	0.8134	7.2175	-2.4866
33 H	-0.8134	7.2175	-2.4866
34 H	0.8134	-7.2175	-2.4866
35 H	-0.8134	-7.2175	-2.4866
36 Cr	0.0000	1.6911	-0.2508
37 Cr	-0.0000	-1.6911	-0.2508
38 Cr	0.0000	-0.0000	1.4197
39 Cr	-1.7971	0.0000	-0.2282
40 Cr	0.0000	0.0000	-1.9001
41 Cr	1.7971	-0.0000	-0.2282
42 C	-0.0000	-4.9378	-1.2632
43 C	0.0000	4.9378	-1.2632
44 C	-0.0000	-6.3963	-0.7933
45 C	0.0000	6.3963	-0.7933

Symmetry C(2V)

Irreducible representations and SCF occupations

A1 61 \ 61
A2 33 \ 33
B1 48 \ 47
B2 44 \ 44
total 371

Spin densities

	Mulliken	mdc m	mdc	mdc q
Cr	-0.06	-0.05	-0.00	-0.01
Cr	-0.06	-0.05	-0.00	-0.01
Cr	-0.15	-0.12	-0.06	-0.06
Cr	0.85	0.77	0.56	0.56
Cr	-0.09	-0.06	-0.02	-0.02
Cr	0.85	0.77	0.56	0.56
Sb	-0.02	-0.01	0.00	-0.00
Sb	-0.02	-0.01	0.00	-0.00
Sb	-0.02	-0.01	0.00	-0.00
Sb	-0.02	-0.01	0.00	-0.00
Sb	-0.03	-0.02	-0.00	-0.00
Sb	-0.03	-0.02	-0.00	-0.00
Sb	-0.03	-0.02	-0.00	-0.00
Sb	-0.03	-0.02	-0.00	-0.00
Sn	0.01	0.01	0.00	0.01
Sn	0.01	0.01	0.00	0.01
Sn	-0.04	-0.03	-0.00	0.00
Sn	-0.04	-0.03	-0.01	-0.00
Sn	-0.01	-0.01	-0.01	-0.01
Sn	-0.01	-0.01	-0.01	-0.01
Sn	-0.04	-0.03	-0.01	-0.00
Sn	-0.04	-0.03	-0.00	0.00
N	0.00	0.00	0.00	0.01
N	0.00	0.00	0.00	0.01
C	-0.00	-0.00	0.00	0.01
H	0.00	-0.00	-0.00	-0.00
H	0.00	-0.00	-0.00	-0.00
C	-0.00	-0.00	0.00	0.01
H	0.00	-0.00	-0.00	-0.00
H	0.00	-0.00	-0.00	-0.00
H	0.00	-0.00	-0.00	-0.00
H	0.00	-0.00	-0.00	-0.00
H	0.00	-0.00	-0.00	-0.00

H	0.00	-0.00	-0.00	-0.00
C	0.00	-0.00	0.00	-0.00
H	-0.00	-0.00	-0.00	0.00
H	-0.00	-0.00	-0.00	0.00
C	0.00	-0.00	0.00	-0.00
H	-0.00	-0.00	-0.00	0.00
H	-0.00	-0.00	-0.00	0.00
N	0.00	0.00	0.00	0.00
H	-0.00	-0.00	-0.00	-0.00
H	-0.00	-0.00	-0.00	-0.00
N	0.00	0.00	0.00	0.00
H	-0.00	-0.00	-0.00	-0.00
H	-0.00	-0.00	-0.00	-0.00

<S2> = 0.88852 exact <S2> = 0.75

(c) Title: **Cr6F2: $^{2}B_{1g}$ state**

Molecular Formula: Cr6F2Sb8Sn8 Charge: -5.0
Job Type: Geometry Optimization

no of valence electrons: 335.0
spin polarization (alpha-beta = Ms): 1.0

no of basis functions: 660

basis set: TZP core: small

frozen cores:
Cr 1s 2s 2p
Sb 1s 2s 2p 3s 3p 3d 4s 4p
Sn 1s 2s 2p 3s 3p 3d 4s 4p
F 1s

functional: PBE

relativistic options: scalar ZORA,MAPA

no symmetry
SCF Energy:
-5.217988 au
-141.9887 eV
-3274.34 kcal/mol
-13699.83 kJ/mol

Technical details of geometry convergence
Optimization method Quasi Newton
required 2 steps to reach convergence

Optimized cartesian coordinates in Angstrom:

0 Sn	4.1672	1.5349	0.0000
1 Sn	4.1672	-1.5349	0.0000
2 Sn	1.5349	-4.1672	0.0000
3 Sn	-1.5349	-4.1672	0.0000
4 Sn	-4.1672	-1.5349	0.0000
5 Sn	-4.1672	1.5349	0.0000
6 Sn	-1.5349	4.1672	0.0000
7 Sn	1.5349	4.1672	0.0000
8 Sb	1.9777	1.9777	1.9584
9 Sb	1.9777	1.9777	-1.9584
10 Sb	1.9777	-1.9777	1.9584
11 Sb	1.9777	-1.9777	-1.9584
12 Sb	-1.9777	-1.9777	-1.9584
13 Sb	-1.9777	-1.9777	1.9584
14 Sb	-1.9777	1.9777	-1.9584
15 Sb	-1.9777	1.9777	1.9584
16 F	0.0000	0.0000	-3.6785
17 F	0.0000	0.0000	3.6785
18 Cr	0.0000	0.0000	1.7026
19 Cr	0.0000	0.0000	-1.7026
20 Cr	1.6923	-0.0000	0.0000
21 Cr	-0.0000	-1.6923	0.0000
22 Cr	-1.6923	0.0000	0.0000
23 Cr	0.0000	1.6923	0.0000

Symmetry D(4H)

Irreducible representations and SCF occupations

A1.g 19 \ 19
A2.g 8 \ 8
B1.g 11 \ 10
B2.g 12 \ 12
E1.g 34 \ 34
A1.u 5 \ 5
A2.u 14 \ 14
B1.u 8 \ 8
B2.u 7 \ 7
E1.u 50 \ 50
total 335

population analysis
Spin densities

	Mulliken	mdc m	mdc	mdc q
Cr	0.11	0.09	0.07	0.07
Cr	0.11	0.09	0.07	0.07
Cr	-0.01	-0.00	-0.00	-0.00
Cr	-0.01	-0.00	-0.00	-0.00
Cr	-0.01	-0.00	-0.00	-0.00
Cr	-0.01	-0.00	-0.00	-0.00
Sb	0.01	0.01	0.02	0.01
Sb	0.01	0.01	0.02	0.01
Sb	0.01	0.01	0.02	0.01
Sb	0.01	0.01	0.02	0.01
Sb	0.01	0.01	0.02	0.01
Sb	0.01	0.01	0.02	0.01
Sb	0.01	0.01	0.02	0.01
Sb	0.01	0.01	0.02	0.01
Sn	0.10	0.09	0.09	0.10
Sn	0.10	0.09	0.09	0.10
Sn	0.10	0.09	0.09	0.10
Sn	0.10	0.09	0.09	0.10
Sn	0.10	0.09	0.09	0.10
Sn	0.10	0.09	0.09	0.10
Sn	0.10	0.09	0.09	0.10
Sn	0.10	0.09	0.09	0.10
F	-0.00	-0.00	0.00	-0.01
F	-0.00	-0.00	0.00	-0.01

<S2> = 0.7537 exact <S2> = 0.75

Frequency/cm-1 Intensity

41.582	102.034
41.650	102.050
43.299	118.696
45.137	120.269
49.877	119.842
50.353	109.625
50.353	109.625
53.840	120.309
71.330	119.997
72.083	93.338
72.735	118.908
74.747	119.407
76.606	106.048
76.648	106.052
76.808	113.136
97.885	108.343
97.927	108.397
103.886	73.445
103.886	73.445
104.357	109.014
109.928	118.951
110.659	96.055
110.695	96.086
119.404	120.493
120.560	116.234
120.560	116.234
121.787	117.541
123.477	120.838
123.632	118.258
125.152	115.356
130.589	85.378
130.753	118.624
131.064	59.136
131.066	59.049
131.207	49.392
131.207	49.392
134.094	117.168
137.145	41.750
137.152	41.702

143.250	120.599
153.713	31.518
153.713	31.518
155.576	119.483
158.181	109.981
158.734	47.012
158.742	47.007
173.448	47.519
184.007	56.322
184.007	56.320
191.492	33.759
222.197	49.461
222.197	49.461
260.786	52.735
263.626	53.667
264.493	49.738
264.494	49.738
283.952	38.132
293.124	52.585
297.219	51.989
297.219	51.989
308.687	52.972
321.623	39.095
340.147	51.989
340.147	51.989
398.430	27.895
415.086	29.677

Thermochemistry

E	-5.217988 au	-141.9887 eV	-3274.34 kcal/mol	-13699.83 kJ/mol
U	-5.148940 au	-140.1098 eV	-3231.01 kcal/mol	-13518.54 kJ/mol
H	-5.147996 au	-140.0841 eV	-3230.42 kcal/mol	-13516.06 kJ/mol
-TS	-0.132667 au	-3.6100 eV	-83.25 kcal/mol	-348.32 kJ/mol
G	-5.280663 au	-143.6941 eV	-3313.67 kcal/mol	-13864.38 kJ/mol

Contributions to entropy (in kcal/mol at T= 298.15 K)

trans	rot	vib	total
14.62	11.06	57.57	83.25

(d) Title: **Cr6F2: $^{2}E_{u}$ state**
Job Type: Geometry Optimization

Molecular Formula: Cr6F2Sb8Sn8 Charge: -5.0

no of valence electrons: 335.0
spin polarization (alpha-beta = Ms): 1.0

no of basis functions: 660
basis set: TZP core: small

frozen cores:
Cr 1s 2s 2p
Sb 1s 2s 2p 3s 3p 3d 4s 4p
Sn 1s 2s 2p 3s 3p 3d 4s 4p
F 1s

functional: PBE

relativistic options: scalar ZORA,MAPA

no symmetry
SCF Energy:
-5.226707 au
-142.2259 eV
-3279.81 kcal/mol
-13722.72 kJ/mol

Technical details of geometry convergence
Optimization method Quasi Newton
required 2 steps to reach convergence

Optimized cartesian coordinates in Angstrom:

0 Sn	-1.5006	0.0000	-4.2170
1 Sn	1.5006	-0.0000	-4.2170
2 Sn	4.1827	0.0000	-1.4734
3 Sn	4.1652	0.0000	1.5192
4 Sn	1.5095	0.0000	4.2149
5 Sn	-1.5095	-0.0000	4.2149
6 Sn	-4.1652	-0.0000	1.5192
7 Sn	-4.1827	-0.0000	-1.4734
8 Sb	-1.9995	1.9328	-2.0061
9 Sb	-1.9995	-1.9328	-2.0061
10 Sb	1.9995	1.9328	-2.0061
11 Sb	1.9995	-1.9328	-2.0061
12 Sb	1.9788	-1.9473	1.9926
13 Sb	1.9788	1.9473	1.9926
14 Sb	-1.9788	-1.9473	1.9926
15 Sb	-1.9788	1.9473	1.9926
16 F	-0.0000	-3.6230	-0.0252
17 F	0.0000	3.6230	-0.0252
18 Cr	-0.0000	1.6702	-0.0313
19 Cr	0.0000	-1.6702	-0.0313
20 Cr	0.0000	-0.0000	-1.7605
21 Cr	1.6801	0.0000	-0.0170
22 Cr	0.0000	0.0000	1.7984
23 Cr	-1.6801	-0.0000	-0.0170

Symmetry C(2V)

Irreducible representations and SCF occupations

A1 55 \ 54
A2 30 \ 30
B1 45 \ 45
B2 38 \ 38
total 335

population analysis

Spin densities

	Mulliken	mdc m	mdc	mdc q
Cr	0.02	0.02	0.04	0.04
Cr	0.02	0.02	0.04	0.04
Cr	0.30	0.27	0.20	0.20

Cr	-0.17	-0.14	-0.08	-0.08
Cr	1.14	1.04	0.75	0.76
Cr	-0.17	-0.14	-0.08	-0.08
Sb	-0.01	-0.01	-0.00	-0.01
Sb	-0.01	-0.01	-0.00	-0.01
Sb	-0.01	-0.01	-0.00	-0.01
Sb	-0.01	-0.01	-0.00	-0.01
Sb	-0.03	-0.02	0.00	-0.00
Sb	-0.03	-0.02	0.00	-0.00
Sb	-0.03	-0.02	0.00	-0.00
Sb	-0.03	-0.02	0.00	-0.00
Sn	0.00	0.00	0.01	0.01
Sn	0.00	0.00	0.01	0.01
Sn	0.00	0.00	0.00	0.00
Sn	0.05	0.04	0.04	0.04
Sn	-0.04	-0.03	0.01	0.01
Sn	-0.04	-0.03	0.01	0.01
Sn	0.05	0.04	0.04	0.04
Sn	0.00	0.00	0.00	0.00
F	0.00	0.00	0.01	0.01
F	0.00	0.00	0.01	0.01

<S2> = 0.84844 exact <S2> = 0.75

Frequency/cm-1	Intensity
36.066	112.021
44.787	120.225
47.039	102.083
47.468	102.011
47.702	102.723
51.722	110.937
54.981	120.219
55.897	118.580
65.116	113.791
69.755	114.290
69.855	93.959
72.719	115.792
75.511	113.624
78.329	113.610
78.675	108.302
94.407	72.331

101.393	81.988
104.467	106.743
105.492	99.109
109.766	104.135
111.727	94.046
119.933	118.344
120.393	104.249
120.696	99.990
122.294	105.366
123.270	115.313
123.674	118.834
125.448	117.264
125.497	97.978
126.228	65.589
126.498	110.868
128.353	95.564
129.723	105.068
129.894	89.637
129.963	59.529
130.875	53.003
135.327	69.409
137.586	42.677
141.387	57.302
143.329	120.537
151.286	34.288
152.399	97.187
154.677	50.183
155.902	28.325
157.072	100.962
158.019	37.784
171.146	47.872
174.278	38.271
184.202	51.415
185.304	48.264
196.567	59.379
212.652	47.436
217.040	48.618
232.474	53.663
232.494	53.703
253.876	53.673
263.226	53.796
272.594	50.345
284.082	49.844
284.260	51.970

297.740	37.140
306.390	51.600
320.584	41.583
335.513	51.721
418.772	27.799
429.343	27.872

Thermochemistry

E	-5.225782 au	-142.2008 eV	-3279.23 kcal/mol	-13720.29 kJ/mol
U	-5.156938 au	-140.3274 eV	-3236.03 kcal/mol	-13539.54 kJ/mol
H	-5.155994 au	-140.3017 eV	-3235.44 kcal/mol	-13537.06 kJ/mol
-TS	-0.134631 au	-3.6635 eV	-84.48 kcal/mol	-353.47 kJ/mol
G	-5.290624 au	-143.9652 eV	-3319.92 kcal/mol	-13890.53 kJ/mol

Contributions to entropy (in kcal/mol at T= 298.15 K)

trans	rot	vib	total
14.62	11.88	57.98	84.48

(e) Title: **Cr6Te8(CO)6**

Molecular Formula: C6Cr6O6Te8 Charge: 0.0
Job Type: Geometry Optimization

no of valence electrons: 136.0

no of basis functions: 612
basis set: TZP core: small

frozen cores:
Cr 1s 2s 2p
Te 1s 2s 2p 3s 3p 3d 4s 4p
C 1s
O 1s

functional: PBE

relativistic options: scalar ZORA,MAPA

no symmetry
SCF Energy:
-6.562279 au
-178.5687 eV
-4117.89 kcal/mol
-17229.26 kJ/mol

Technical details of geometry convergence
Optimization method Quasi Newton
required 14 steps to reach convergence

Optimized cartesian coordinates in Angstrom:

0	Te	1.8529	1.8529	-1.8529
1	Te	-1.8529	1.8529	-1.8529
2	Te	1.8529	-1.8529	-1.8529
3	Te	-1.8529	-1.8529	-1.8529
4	Te	-1.8529	-1.8529	1.8529
5	Te	1.8529	-1.8529	1.8529
6	Te	-1.8529	1.8529	1.8529

7 Te	1.8529	1.8529	1.8529
8 O	0.0000	-4.8892	0.0000
9 O	0.0000	0.0000	4.8892
10 O	-4.8892	0.0000	0.0000
11 O	0.0000	4.8892	0.0000
12 O	0.0000	0.0000	-4.8892
13 O	4.8892	0.0000	0.0000
14 Cr	1.8471	0.0000	0.0000
15 Cr	-1.8471	0.0000	0.0000
16 Cr	0.0000	0.0000	-1.8471
17 Cr	0.0000	0.0000	1.8471
18 Cr	0.0000	1.8471	0.0000
19 Cr	0.0000	-1.8471	0.0000
20 C	3.7182	0.0000	0.0000
21 C	-3.7182	0.0000	0.0000
22 C	0.0000	3.7182	0.0000
23 C	0.0000	0.0000	-3.7182
24 C	0.0000	-3.7182	0.0000
25 C	0.0000	0.0000	3.7182

Symmetry O(H)

Irreducible representations and SCF occupations

A1.g 18
E.g 32
T1.g 30
T2.g 54
A2.u 6
E.u 12
T2.u 36
T1.u 84
total 272

(f) Title: **Model 2**

Molecular Formula: Cr6F2Sb8Sn8 Charge: -5.0
Job Type: Geometry Optimization

no of valence electrons: 335.0
spin polarization (alpha-beta = Ms): 1.0

no of basis functions: 660
basis set: TZP   core: small

frozen cores:
Cr 1s 2s 2p
Sn 1s 2s 2p 3s 3p 3d 4s 4p
Sb 1s 2s 2p 3s 3p 3d 4s 4p
F 1s

functional: PBE

relativistic options: scalar ZORA,MAPA

no symmetry
SCF Energy:
-5.193272 au
-141.3161 eV
-3258.83 kcal/mol
-13634.93 kJ/mol

Technical details of geometry convergence
Optimization method Quasi Newton
required 16 steps to reach convergence

Optimized cartesian coordinates in Angstrom:

0 Sn	-2.0191	2.0685	-2.0165
1 Sn	-2.0191	-2.0685	-2.0165
2 Sn	2.0191	2.0685	-2.0165
3 Sn	2.0191	-2.0685	-2.0165
4 Sn	2.0285	-2.0649	2.0101

5 Sn	2.0285	2.0649	2.0101
6 Sn	-2.0285	-2.0649	2.0101
7 Sn	-2.0285	2.0649	2.0101
8 Sb	-1.4882	-0.0000	-4.1245
9 Sb	1.4882	0.0000	-4.1245
10 Sb	4.0937	0.0000	-1.4868
11 Sb	4.1029	0.0000	1.4649
12 Sb	1.4888	0.0000	4.1216
13 Sb	-1.4888	-0.0000	4.1216
14 Sb	-4.1029	-0.0000	1.4649
15 Sb	-4.0937	-0.0000	-1.4868
16 F	0.0000	-3.5378	0.0012
17 F	-0.0000	3.5378	0.0012
18 Cr	-0.0000	1.5931	0.0021
19 Cr	0.0000	-1.5931	0.0021
20 Cr	0.0000	-0.0000	-1.7835
21 Cr	1.6843	-0.0000	-0.0051
22 Cr	0.0000	0.0000	1.7867
23 Cr	-1.6843	0.0000	-0.0051

Symmetry C(2V)

Irreducible representations and SCF occupations

A1 55 \ 54
A2 30 \ 30
B1 45 \ 45
B2 38 \ 38
total 335

Spin densities

	Mulliken	mdc m	mdc	mdc q
Cr	-0.10	-0.07	-0.03	-0.03
Cr	-0.10	-0.07	-0.03	-0.03
Cr	1.04	0.92	0.69	0.69
Cr	-0.32	-0.27	-0.17	-0.18
Cr	1.09	0.97	0.72	0.73
Cr	-0.32	-0.27	-0.17	-0.18
Sn	-0.02	-0.02	-0.00	-0.01
Sn	-0.02	-0.02	-0.00	-0.01
Sn	-0.02	-0.02	-0.00	-0.01
Sn	-0.02	-0.02	-0.00	-0.01

Sn	-0.02	-0.02	-0.01	-0.01
Sn	-0.02	-0.02	-0.01	-0.01
Sn	-0.02	-0.02	-0.01	-0.01
Sn	-0.02	-0.02	-0.01	-0.01
Sb	-0.02	-0.02	0.02	0.02
Sb	-0.02	-0.02	0.02	0.02
Sb	0.00	-0.00	-0.01	-0.01
Sb	0.00	-0.00	-0.01	-0.01
Sb	-0.02	-0.02	0.02	0.02
Sb	-0.02	-0.02	0.02	0.02
Sb	0.00	-0.00	-0.01	-0.01
Sb	0.00	-0.00	-0.01	-0.01
F	-0.00	-0.00	-0.01	0.00
F	-0.00	-0.00	-0.01	0.00

<S2> = 0.98848 exact <S2> = 0.75

(g) Title: **Model 3**

Molecular Formula: Cr6F2Sb8Sn8 Charge: -5.0
Job Type: Geometry Optimization
Technical details of geometry convergence
Optimization method Quasi Newton
required 17 steps to reach convergence

no of basis functions: 660

basis set: TZP core: small

frozen cores:
Cr 1s 2s 2p
Sn 1s 2s 2p 3s 3p 3d 4s 4p
Sb 1s 2s 2p 3s 3p 3d 4s 4p
F 1s

no of valence electrons: 335.0
spin polarization (alpha-beta = Ms): 1.0

functional: PBE

relativistic options: scalar ZORA,MAPA

no symmetry
SCF Energy: -5.160519 au -140.4249 eV -3238.27 kcal/mol -13548.94 kJ/mol

Optimized cartesian coordinates in Angstrom:

0	Sn	2.5199	-1.3582	2.0516
1	Sn	-1.3486	-2.5191	-2.0419
2	Sn	-2.5199	1.3582	2.0516
3	Sn	1.3486	2.5191	-2.0419
4	Sn	-0.2838	-4.4874	-0.0306
5	Sn	-4.4597	0.2949	0.0424
6	Sn	0.2838	4.4874	-0.0306
7	Sn	4.4597	-0.2949	0.0424
8	Sb	2.4903	-1.2969	-1.9408
9	Sb	-1.3044	-2.4744	1.9319
10	Sb	-2.4903	1.2969	-1.9408
11	Sb	1.3044	2.4744	1.9319
12	Sb	2.5315	-3.5700	-0.0114

13 Sb	-3.5391	-2.4983	0.0042
14 Sb	-2.5315	3.5700	-0.0114
15 Sb	3.5391	2.4983	0.0042
16 F	0.0000	-0.0000	-3.6104
17 F	0.0000	-0.0000	3.6078
18 Cr	0.0000	-0.0000	1.6601
19 Cr	-0.0000	0.0000	-1.6641
20 Cr	0.5079	-1.7018	-0.0036
21 Cr	-1.6066	-0.4555	0.0016
22 Cr	-0.5079	1.7018	-0.0036
23 Cr	1.6066	0.4555	0.0016

Symmetry C(2)

Irreducible representations and SCF occupations

A 84 \ 84
B 84 \ 83
total 335

Spin densities

	Mulliken	mdc m	mdc	mdc q
Cr	0.01	0.01	0.04	0.04
Cr	0.04	0.05	0.07	0.07
Cr	0.88	0.79	0.59	0.59
Cr	-0.29	-0.24	-0.14	-0.15
Cr	0.88	0.79	0.59	0.59
Cr	-0.29	-0.24	-0.14	-0.15
Sn	-0.02	-0.01	-0.00	-0.01
Sb	-0.02	-0.01	-0.00	-0.00
Sb	-0.03	-0.02	-0.01	-0.01
Sn	-0.03	-0.02	-0.01	-0.01
Sb	-0.02	-0.01	-0.00	-0.00
Sn	-0.02	-0.01	-0.00	-0.01
Sn	-0.03	-0.02	-0.01	-0.01
Sb	-0.03	-0.02	-0.01	-0.01
Sb	-0.02	-0.01	0.02	0.02
Sn	-0.02	-0.02	0.01	0.01
Sb	-0.00	-0.00	-0.01	-0.01
Sn	0.02	0.01	0.01	0.02
Sb	-0.02	-0.01	0.02	0.02
Sn	-0.02	-0.02	0.01	0.01
Sb	-0.00	-0.00	-0.01	-0.01

Sn	0.02	0.01	0.01	0.02
F	0.00	0.00	0.01	0.01
F	0.00	0.00	0.01	0.01

<S2> = 0.91791 exact <S2> = 0.75

Frequency/cm-1	Intensity
-113.756	55.481
28.179	111.941
42.753	120.060
44.855	97.886
47.887	107.202
49.936	103.385
52.206	99.561
55.122	117.721
56.112	112.455
59.277	114.727
64.123	97.680
67.269	109.775
70.445	118.062
76.534	110.900
77.504	119.399
79.618	109.346
88.959	81.085
93.789	91.376
99.683	99.654
103.923	102.791
104.534	116.276
109.314	110.579
111.133	113.593
114.540	102.201
118.490	92.427
118.777	114.461
119.415	106.701
123.043	78.818
123.598	97.127
125.677	71.771
127.291	115.765
128.847	84.121
129.389	112.744
130.458	43.075

131.310	55.011
132.510	68.022
132.908	110.102
134.717	115.932
135.704	33.637
138.799	44.060
138.857	118.861
142.412	38.908
146.896	98.095
147.436	40.207
153.686	112.270
154.158	33.261
155.651	42.140
162.155	52.075
177.986	48.791
180.410	58.652
183.016	44.008
185.410	61.704
205.840	50.075
206.817	48.002
218.367	55.395
224.603	51.536
254.903	53.477
257.491	51.226
269.158	50.724
273.146	53.294
296.004	51.659
297.495	36.514
321.331	39.960
328.702	51.651
420.568	27.623
431.593	27.697

Thermochemistry

E	-5.160519 au	-140.4249 eV	-3238.27 kcal/mol	-13548.94 kJ/mol
U	-5.092879 au	-138.5843 eV	-3195.83 kcal/mol	-13371.35 kJ/mol
H	-5.091935 au	-138.5586 eV	-3195.24 kcal/mol	-13368.87 kJ/mol
-TS	-0.135358 au	-3.6833 eV	-84.94 kcal/mol	-355.38 kJ/mol
G	-5.227293 au	-142.2419 eV	-3280.18 kcal/mol	-13724.26 kJ/mol

Contributions to entropy (in kcal/mol at T= 298.15 K)

trans	rot	vib	total
14.62	11.88	58.44	84.94

(h) Title: **Model 4**

Molecular Formula: Cr6F2Sb8Sn8 Charge: -5.0
Job Type: Geometry Optimization
Technical details of geometry convergence
Optimization method Quasi Newton
required 39 steps to reach convergence

no of valence electrons: 335.0
spin polarization (alpha-beta = Ms): 1.0

no of basis functions: 660
basis set: TZP core: small

frozen cores:
Cr 1s 2s 2p
Sn 1s 2s 2p 3s 3p 3d 4s 4p
Sb 1s 2s 2p 3s 3p 3d 4s 4p
F 1s

functional: PBE

relativistic options: scalar ZORA,MAPA

no symmetry
SCF Energy:
-5.213527 au
-141.8673 eV
-3271.54 kcal/mol
-13688.11 kJ/mol

Optimized cartesian coordinates in Angstrom:

0	Sn	2.0263	2.0494	2.0530
1	Sn	4.2181	-1.5366	-0.0124

2 Sn	1.5050	-4.1889	-0.0007
3 Sn	-1.4895	-4.2086	-0.0054
4 Sn	-4.2181	-1.4962	-0.0048
5 Sn	-4.2041	1.5075	-0.0006
6 Sn	-1.4788	4.1865	-0.0240
7 Sn	1.4767	4.1928	0.0208
8 Sb	1.9759	2.0288	-1.9498
9 Sb	2.0000	-1.9890	1.9365
10 Sb	1.9997	-1.9915	-1.9382
11 Sb	-2.0120	-2.0113	-1.9403
12 Sb	-2.0078	-2.0092	1.9269
13 Sb	-1.9900	1.9905	-1.9469
14 Sb	-1.9772	1.9972	1.9303
15 Sb	4.1386	1.4545	-0.0195
16 F	0.0170	-0.0088	-3.6188
17 F	0.0423	0.0288	3.5981
18 Cr	0.0102	-0.0273	1.6492
19 Cr	-0.0048	-0.0360	-1.6659
20 Cr	1.7527	0.0646	0.0215
21 Cr	-0.0300	-1.6933	-0.0072
22 Cr	-1.7681	0.0069	-0.0046
23 Cr	0.0178	1.6891	0.0027

Symmetry NOSYM

Irreducible representations and SCF occupations

A 168 \ 167
total 335

Spin densities

	Mulliken	mdc m	mdc	mdc q
Cr	0.04	0.04	0.05	0.05
Cr	0.10	0.10	0.10	0.10
Cr	0.80	0.73	0.55	0.55
Cr	-0.08	-0.06	-0.02	-0.02
Cr	0.42	0.38	0.28	0.28
Cr	-0.27	-0.23	-0.15	-0.15
Sn	0.02	0.02	0.03	0.02
Sb	-0.01	-0.01	0.00	-0.00
Sb	-0.02	-0.02	-0.00	-0.00
Sb	-0.03	-0.02	-0.00	-0.00
Sb	-0.02	-0.01	-0.00	-0.00

Sb	-0.02	-0.01	-0.01	-0.01
Sb	-0.00	0.00	0.01	0.00
Sb	0.00	0.00	0.01	-0.00
Sb	-0.02	-0.01	0.01	0.01
Sn	-0.01	-0.01	0.01	0.01
Sn	-0.00	-0.00	-0.01	-0.01
Sn	-0.01	-0.01	-0.01	-0.01
Sn	-0.02	-0.01	0.00	0.00
Sn	-0.00	0.00	0.01	0.01
Sn	0.03	0.03	0.03	0.04
Sn	0.10	0.08	0.09	0.10
F	0.00	0.01	0.01	0.01
F	0.00	0.01	0.01	0.02

<S2> = 0.83004 exact <S2> = 0.75

----

5. Supplementary References

[1] G. M. Sheldrick, *Acta Cryst, Sect. A: Fund. Adv*. **2015**, *71*, 3−8.

[2] O. V. Dolomanov, L. J. Bourhis, R. J. Gildea, J. A. K. Howard, H. Puschmann, *J. Appl. Crystallog*r. **2009**, *42*, 339−341.
